# HIV-protease inhibitors potentiate the activity of carfilzomib in triple-negative breast cancer

**DOI:** 10.1038/s41416-024-02774-9

**Published:** 2024-07-05

**Authors:** Andrej Besse, Lenka Sedlarikova, Lorina Buechler, Marianne Kraus, Chieh-Hsiang Yang, Nicol Strakova, Karel Soucek, Jiri Navratil, Marek Svoboda, Alana L. Welm, Markus Joerger, Christoph Driessen, Lenka Besse

**Affiliations:** 1 Laboratory of Experimental Oncology, Department of Oncology and Hematology, Cantonal Hospital St. Gallen, St. Gallen, 9000 Switzerland; 2 Department of Biology, Faculty of Medicine, Masaryk University, Brno, 62500 Czech Republic; 3 Babak Myeloma Group, Department of Pathological Physiology, Masaryk University, Brno, 62500 Czech Republic; 4 Department of Oncological Sciences, University of Utah, Salt Lake City, UT USA; 5 Huntsman Cancer Institute, University of Utah, Salt Lake City, UT USA; 6 Department of Cytokinetics, Institute of Biophysics of the Czech Academy of Sciences, Brno, 612 00 Czech Republic; 7 International Clinical Research Center, St. Anne’s University Hospital Brno, Brno, Czech Republic; 8 Department of Comprehensive Cancer Care, Masaryk Memorial Cancer Institute, Brno, 62500 Czech Republic; 9 Faculty of Medicine, Masaryk University, Brno, 62500 Czech Republic; 10 Department of Oncology and Hematology, Cantonal Hospital St. Gallen, St. Gallen, 9000 Switzerland; 11 Present Address: Department of Biology, Faculty of Medicine, Masaryk University, Brno, 62500 Czech Republic; 12 Present Address: Veterinary Research Institute, Brno, 62500 Czech Republic

**Keywords:** Breast cancer, Cancer therapeutic resistance

## Abstract

**Background:**

Resistance to chemotherapy is a major problem in the treatment of patients with triple-negative breast cancer (TNBC). Preclinical data suggest that TNBC is dependent on proteasomes; however, clinical observations indicate that the efficacy of proteasome inhibitors in TNBC may be limited, suggesting the need for combination therapies.

**Methods:**

We compared bortezomib and carfilzomib and their combinations with nelfinavir and lopinavir in TNBC cell lines and primary cells with regard to their cytotoxic activity, functional proteasome inhibition, and induction of the unfolded protein response (UPR). Furthermore, we evaluated the involvement of sXBP1, ABCB1, and ABCG2 in the cytotoxic activity of drug combinations.

**Results:**

Carfilzomib, via proteasome β5 + β2 inhibition, is more cytotoxic in TNBC than bortezomib, which inhibits β5 + β1 proteasome subunits. The cytotoxicity of carfilzomib was significantly potentiated by nelfinavir or lopinavir. Carfilzomib with lopinavir induced endoplasmic reticulum stress and pro-apoptotic UPR through the accumulation of excess proteasomal substrate protein in TNBC in vitro. Moreover, lopinavir increased the intracellular availability of carfilzomib by inhibiting carfilzomib export from cells that express high levels and activity of ABCB1, but not ABCG2.

**Conclusion:**

Proteasome inhibition by carfilzomib combined with nelfinavir/lopinavir represents a potential treatment option for TNBC, warranting further investigation.

## Introduction

Malignant breast cancer cells are characterized by intrinsic genomic instability, leading to the accumulation of misfolded proteins. Adequate protein quality control capacity to maintain misfolded proteins is critical for their survival. Therefore, malignant cells increase the amount and activity of molecular chaperones to enhance global protein folding and support oncogenic processes [1]. At the same time, the ability to degrade misfolded proteins is significantly enhanced during malignant transformation and as a consequence of increased breast cancer aggressiveness via the Nuclear Respiratory Factor 1(NRF1) and Nuclear Factor Erythroid 2-Related Factor 2 (NRF2) transcription programs, to avoid detrimental effects of misfolded proteins [2]. Similarly, the most aggressive subtype of breast cancer with the poorest prognosis, basal-like triple-negative breast cancer (TNBC), is strongly dependent on proper proteasome function [3]. Proteasome inhibition in basal-like TNBC is selectively cytotoxic in vitro, reduces the growth of established basal-like TNBC tumors in mice and blocks tumor initiation [3].

The proteasome is a multi-catalytic protein complex with three individual proteolytic β-subunits located at the core of the proteasome with distinct substrate specificity and activity (β5, chymotrypsin-like; β2, trypsin-like; β1, caspase-like) [4]. The β5 subunit of the proteasome was initially identified as the rate-limiting protease for proteasomal protein turnover based on the individual genetic knockdown of the proteolytically active protein domains of the constitutive proteasome in yeast [5–7]. Accordingly, all clinically available proteasome inhibitors (PIs) by design target the β5 proteasome subunit. Recent evidence has shown that all β5-targeting PIs lose their subunit selectivity at higher concentrations and co-inhibit either β1 and/or β2 type proteasome subunits. These co-inhibition patterns differed between the individual approved PIs: boronate-based PI bortezomib and ixazomib showed β5 + β1 inhibition, whereas epoxyketone-based PI carfilzomib showed a β5 + β2 inhibition pattern. Consequently, co-inhibition of the β2 proteasome subunit together with the β5 subunit translates into stronger proteasome inhibition and cytotoxicity [8, 9], with carfilzomib being the only U.S. Food and Drug Administration (FDA)-approved PI showing β2 co-inhibitory activity at higher doses.

Clinically relevant doses of approved PIs induce selective β5 subunit inhibition in TNBC and are, therefore, not cytotoxic. However, specific β2, but not β1, proteasome inhibition combined with bortezomib or carfilzomib increases the cytotoxicity of both drugs [10]. More effective proteasome inhibition provided by β5 and β2 inhibition leads to stronger proteasome substrate accumulation and suppression of NRF1-regulated induction of proteasome synthesis in basal-like TNBC [8, 10]. However, the clinically available options to increase the efficacy of currently available PIs towards the most effective β5 + β2 proteasome inhibition pattern are limited. Recently, the FDA-approved HIV-protease inhibitors (HIV-PIs) nelfinavir and lopinavir were shown to increase the activity of PIs in PI-resistant multiple myeloma (MM) via the induction of the unfolded protein response (UPR) and via multi-drug-transporter ABCB1 inhibition [11, 12]. Importantly, high UPR activity is a hallmark of basal-like TNBC, driving tumorigenesis [13] and ABCB1 inhibition increases the efficacy of PIs in basal-like TNBC [14], suggesting the therapeutic potential of HIV-PIs in TNBC. Moreover, the key UPR sensor Endoplasmic Reticulum To Nucleus Signaling 1(ERN orIRE1α) resides in the ER and deploys a cytoplasmic kinase-endoribonuclease module to activate transcription factor X-Box Binding Protein 1 (XBP1), which facilitates endoplasmic reticulum (ER)-mediated protein folding. Studies in TNBC implicate XBP1-spliced (sXBP1) in promoting tumor vascularization and progression [13], and the XBP1 gene signature is predictive of the survival of patients with TNBC [15]. Further, TNBC cells critically rely on IRE1α to adapt their ER to in vivo stress and adjust the tumor microenvironment to facilitate malignant growth. TNBC reliance on IRE1α is an important vulnerability that can be uniquely exploited as a promising new biological approach to combat this lethal disease [16].

Here, we aimed to analyze whether the dependency on functional proteasome in TNBC makes TNBC more vulnerable to meaningful functional proteasome inhibition and whether the cytotoxic activity of such proteasome inhibition can be potentiated by co-treatment with the UPR-inducing FDA-approved anti-HIV drugs nelfinavir and lopinavir.

## Material And Methods

### Cell lines and chemicals

Basal-like (triple negative: MDA-MB-231, BT549) and luminal (ER + PR+: MCF-7; ER + PR+ Her2+: BT474) cell lines were obtained from commercial sources (American Type Culture Collection, ATCC; Deutsche Sammlung von Mikroorganismen und Zellkulturen, DSMZ) and maintained under standard conditions in RPMI-1640 medium (Merck/Sigma-Aldrich, Buchs, Switzerland) supplemented with 10% heat-inactivated fetal bovine serum (FBS), 100 µg/ml streptomycin, and 100 U/ml penicillin (Merck/Sigma-Aldrich, Buchs, Switzerland). All cell lines were used at the passages under 20, routinely tested for mycoplasma contamination, and authenticated using STR profiling.

The concentrations of PIs bortezomib and carfilzomib, HIV-PIs nelfinavir and lopinavir, and PgP inhibitor reserpine (Med Chem Express, Monmouth Junction, NJ, USA) are specified in the relevant sections.

### Tissue samples and primary cells

Tissue specimens were obtained during standard surgical procedures from patients diagnosed with triple-negative breast cancer who underwent surgery at the Masaryk Memorial Cancer Institute, Brno, Czechia. The basic characteristic of patients is included in Supplementary Table S1. Mechanical disaggregation of tumor specimens was used to obtain viable tumor cells, which were subsequently cultivated as previously described [17]. Written informed consent was obtained from all patients.

### Viability assay

Cell viability was determined by using CellTiter 96® AQueous One Solution or CellTiter-Glo (Promega, Madison, WI, USA). A detailed description is provided in Supplementary Methods.

The coefficient of drug interaction (CDI) was calculated as follows: CDI = AB/(A × B). According to the viability of each group, AB is the ratio of the viability of the combination group to that of the control group and A or B is the ratio of the viability of the single-agent group to that of the control group.

### PDX-derived organoids culture and three-dimensional synergy drug testing

TNBC PDX-derived organoids (PDxOs: HCI-002, HCI-010, and HCI-023) were obtained and cultured as previously described [18, 19]. Written informed consent was obtained from all patients. Matured organoids (~70 μm in diameter) were seeded per well in 384-well tissue culture plates (PerkinElmer, Waltham, MA, USA), and treated with an eight-point (nelfinavir) x eleven-point (carfilzomib) serial dilution in technical quadruplicate, The dosed PDxO plates were covered with Breathe-Easy seals (USA Scientific, Ocala, FL, USA) and incubated for 144 h at 37 °C and 5% CO2. After incubation, the seals were removed, and 15 μl of CellTiterGlo 3D (Promega, Madison, WI, USA) was added to each well. Raw luminescent values from each condition were divided by the values from the untreated wells to obtain viability. Synergy maps and scores were generated using SynergyFinder+ [20, 21]. A more detailed description is provided in Supplementary Methods.

### RNA isolation and qPCR

Total RNA was isolated from cell lines using TRIzol (Thermo Fisher Scientific, Waltham, MA, USA; Thermo Fisher) and Direct-zol RNA MiniPrep (Zymo Research, Irvine, CA, USA). 500 ng of total RNA was reverse transcribed using a High-capacity cDNA Reverse Transcription kit (Thermo Fisher) according to the manufacturer’s recommendations. Subsequently, 10 ng of cDNA was used for qPCR reactions with 2x LightCycler® 480 SYBR Green I Master Mix (Roche, Basel, Switzerland; Roche) or TaqMan Gene Expression Master Mix (Thermo Fisher) on a Light Cycler II instrument (Roche). Primers for SYBR green detection and TaqMan primer/probe mixes (all from Thermo Fisher) are specified in Supplementary data (Supplementary Table S2).

### Activity-based proteasome probes (ABP) labelling

Briefly, the cells were incubated with lopinavir, carfilzomib, or their combination for 1 h. For the assessment of proteasome activity after 1 h, cells were directly harvested for protein isolation, and for the assessment of proteasome activity after 8 h, the medium was replaced with a drug-free medium or medium containing lopinavir, and then cells were harvested for protein isolation. The activity of proteasome subunits was assessed using the recently developed set of subunit-selective activity-based probes (ABP) that differentially visualize individual activities of β1, β2, and β5 subunits of the constitutive and immunoproteasomes [22].

### Western blot

Western blotting was performed as described previously [23] with the following antibodies: anti-ABCB1 (#9126, Cell Signaling Technology, Danvers, MA, USA; CST), anti-ABCG2 (#42078, CST), anti-BIP (GRP78; #610979; BD Biosciences, San Diego, CA, USA; BD), anti-IRE1 (#3294, CST), anti-NOXA (#OP180; Calbiochem/EMD Millipore, MA, USA), anti-PDI (#610946; BD), anti-polyUb (PW 8805-0500; Enzo Life Sciences; Lausen, Switzerland), and anti-sXBP1 (#12782; CST). Anti-β-actin (#8457, CST) and anti-GAPDH POD (Merck/Sigma-Aldrich, Buchs, Switzerland; Merck) were used as loading controls.

### Generation of cells expressing Ub^G76V^-GFP, ABCB1, MERO-GFP and BIP-mGFP and CRISPR/Cas9 knock-outs

A detailed description is provided in Supplementary Methods.

### Flow cytometry

#### Apoptosis determination

MDA-MB-231 cells were exposed to the indicated drugs for 1 h, followed by removal of the drugs and subsequent incubation with 10 µM lopinavir or drug-free medium for 48 h. Cells were stained using the Annexin V/FITC Detection Kit (Vazyme Biotech, Nanjing, China) according to the manufacturer’s instructions and analyzed using FACS Canto II (BD).

#### ABCB1 functional assay by MitoTracker Green FM

Cells were pretreated with lopinavir or reserpine for 2 h and subsequently incubated with MitoTracker Green FM (Thermo Fisher) for 20 min at 37 °C in the dark. Cells were washed and harvested, and green fluorescence intensity was examined using FACS Canto II (BD).

#### Assessment of Ub^G76V^-GFP accumulation

MDA-MB-231_Ub^G76V^-GFP equipped cells were seeded 24 h prior to analysis, and treated with various drugs and combinations to show functional proteasome inhibition. Eight hours post-treatment, the cells were washed, and harvested, and green fluorescence was examined by FACS Canto II (BD).

#### Assessment of functional protein folding using MERO-GFP-equipped cells

MERO-GFP positive cells were seeded 24 h prior to the experiment, and the fluorescence of folded and unfolded GFP was evaluated using a BD Fortessa flow cytometer (BD) at 405 and 488 nm, respectively.

#### Data analysis

Flow cytometry data were analyzed using FlowJo v10 software (BD).

### Assessment of BIP mobility

The BiP-mGFP-equipped cells were seeded 24 h prior to the experiment in a 35 mm µ-dish, and on the following day, FRAP experiments were performed on an LSM700 equipped with a Plan-Apochromat 63x/1.40 Oil Ph3 M27 objective (Zeiss, Oberkochen, Germany). Two images were acquired: prior to bleaching and after the region of interest was bleached with five iterations at maximal laser intensity, followed by the acquisition of 29 frames at 1.1 s intervals post-bleaching.

MDA-MB-231_BiP-mGFP cells were used for further experiments. There the cells were seeded 24 h prior to the experiment in µ-dish 35 mm and the following day the cells were treated with 100 nM carfilzomib, 10 µM lopinavir, and their combination or equal volume of DMSO for 1 h. Afterwards, FRAP experiments were performed as described above.

### Statistical analysis

Relative quantification of raw qPCR data was performed using the 2^-ddCt^ method, and the data were normalized to the housekeeping gene (GAPDH). The kinetics of UPR activation data were normalized to GAPDH and the time point at the end of the 1 h pulse for all treatments and combinations. The values obtained were then normalized to those of untreated cells at all of the time points, which served as a baseline. Unless specified otherwise, for all experiments, data are presented as the mean ± SD of three independent experiments, and flow cytometry data are presented as the median of fluorescence ± SD of at least three independent experiments. Group comparison for continuous data of qPCR was performed with two-way ANOVA with Bonferroni’s post-test; for mean comparison of continuous data, paired or unpaired two-sided *t*-test or one-way ANOVA and Tukey’s or Dennett’s post-test was used. IC_50_ values were obtained using nonlinear regression curve fit analysis. Statistical significance was set at *p* < 0.05. Data were statistically analyzed using GraphPad Prism v8 (GraphPad Software, MA, Boston, USA).

## Results

### Basal-like TNBC cells show increased UPR activation status not associated with decreased folding capacity

**Fig. 1:**
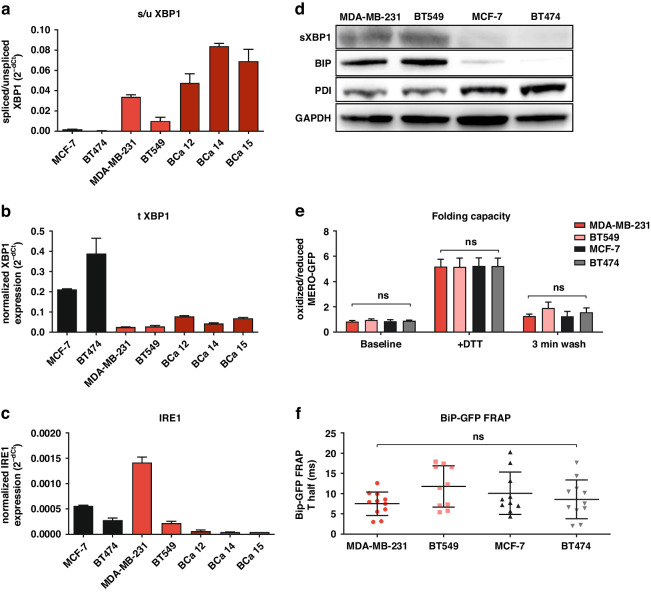
UPR activation status and folding capacity in breast cancer. **a** Basal state of spliced XBP1 (sXBP1) in cell lines and patient-derived primary cells evaluated by qPCR, assessed as a ratio of spliced vs unspliced XBP1 and normalized to GAPDH, which served as a housekeeping gene. The data represent the mean ± SD from 3 independent experiments. **b** Basal state of total XBP1 (tXBP1) in cell lines and patient-derived primary cells evaluated by qPCR and normalized to GAPDH, which served as a housekeeping gene. The data represent the mean ± SD from 3 independent experiments. **c** Basal state of IRE1 in cell lines and patient-derived primary cells evaluated by qPCR and normalized to GAPDH, which served as a housekeeping gene. The data represent the mean ± SD from 3 independent experiments. **d** Basal state of proteins involved in the UPR and protein folding. Western blot analysis was performed with TNBC (MDA-MB-231 and BT549) and non-TNBC (MCF-7 and BT474) cells. Representative images of three independent experiments are shown. GAPDH served as an internal loading control. **e** Ratio of oxidized vs reduced MERO-GFP in the cell lines evaluated by flow cytometry at baseline, immediately after the exposure to DTT and 3 min after washing DTT away. The data represent the mean ± SD from 3 independent experiments. **f** BiP-GFP FRAP analysis of the cell lines at the basal state. The data represent the mean ± SD of T half (in ms) recovery of BiP-GFP fluorescence, evaluated in the individual cells in two independent experiments. BiP-GFP Binding immunoglobulin Protein green-fluorescent protein, DTT dithiothreitol, GAPDH Glyceraldehyde-3-Phosphate Dehydrogenase, IRE1 Inositol-Requiring Enzyme 1, MERO-GFP Mammalian endoplasmic reticulum-localized redox-sensitive green-fluorescent protein, PDI protein disulfide isomerase, XBP1 X-Box-Binding Protein 1.

TNBC cells are characterized by a state of higher basal ER stress, more abundant and dilated ER, and higher levels of sXBP1 [13]. Our data confirmed these findings and showed that TNBC cell lines MDA-MB-231 and BT549 as well as TNBC patient-derived primary cells have significantly higher levels of sXBP1, but lower levels of total XBP1 compared to the luminal cell lines MCF-7 and BT474 (Fig. 1a, b). Interestingly, the levels of IRE1, a transmembrane endoribonuclease that splices XBP1 [24], varied between cells and did not reflect high sXBP1 levels in TNBC cells (Fig. 1c). Cells with higher levels of spliced XBP1 showed high levels of chaperone BIP (also known as ER-luminal 78 kDa glucose-regulated protein, Grp78, HSPA5, or HSP70) (Fig. 1d). BIP plays a central role in UPR activation as it binds to misfolded proteins, and its excess decreases the UPR to chronic, submaximal activation, and to pro-survival UPR response [25]. At the same time, basal-like TNBC cells showed lower levels of protein disulfide isomerase (PDI), which is important for proper protein folding and redox homeostasis (Fig. 1a). Functionally, using the MERO-GFP construct [26] and BiP-GFP FRAP [27] in the ER, we showed that TNBC cells fold proteins equally effectively in comparison to non-TNBC cells (Fig. 1e), and that the mobility of BIP did not significantly differ between TNBC and non-TNBC cells (Fig. 1f). This suggests that the state of higher basal ER stress in basal-like TNBC, represented by increased sXBP1, is not a consequence of less effective protein folding per se but is more likely caused by other biological processes triggering XBP1 splicing [28].

### HIV-protease inhibitors potentiate cytotoxic effects of bortezomib and carfilzomib in TNBC cells with high sXBP1

**Fig. 2:**
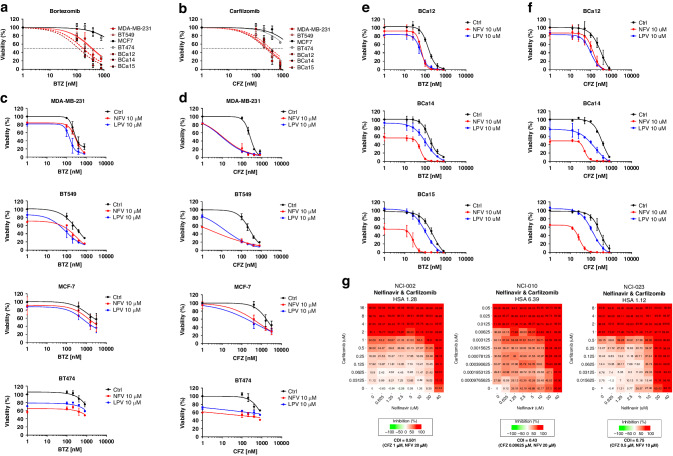
Response of breast cancer cell lines, primary cells and PDX-organoids to bortezomib/carfilzomib alone or in combination with nelfinavir/lopinavir. TNBC cell lines MDA-MB-231 and BT549, non-TNBC cell lines MCF-7 and BT474, and TNBC-primary cells were treated for 1 h with increasing doses of bortezomib (**a**) or carfilzomib (**b**). Subsequently, the cells were placed in a drug-free medium for the next 48 h. The data represent the mean ± SD of 3 independent experiments. Cell lines were treated for 1 h with increasing doses of bortezomib (**c**) or carfilzomib (**d**). Subsequently, the cells were placed in a drug-free medium or incubated with 10 µM nelfinavir or lopinavir for the next 48 h. The data represent the mean ± SD of 3 independent experiments. Viability at the selected time point at which CDI was calculated is depicted in Fig. S1, S2. TNBC patient-derived cells were treated for 1 h with increasing doses of bortezomib (**e**) or carfilzomib (**f**). Subsequently, the cells were placed in a drug-free medium or incubated with 10 µM nelfinavir or lopinavir for the next 48 h. Cell viability was measured 48 h after treatment, and the data represent the mean ± SD of 2 independent experiments. Viability at a selected time point at which CDI was calculated is depicted in Fig. S3, S4. In all experiments, the corresponding IC_50_ values were determined from the dose-response curves and are presented in Supplementary Table S3. **g** Cytotoxicity of carfilzomib and nelfinavir in the three PDxOs. Numbers represent the percentage of growth inhibition. Drug synergy was modelled using SynergyFinder+, CDI of the most synergistic drug combination is presented. BTZ bortezomib, CFZ carfilzomib, LPV lopinavir, NFV nelfinavir.

PI bortezomib and carfilzomib at higher doses differ in their selectivity towards proteasome proteolytic subunits; while bortezomib inhibits β5 and β1 subunits, carfilzomib shows β5 and β2 inhibitory profiles [8]. The sensitivity of MM cells to bortezomib correlates with the level of sXBP1 but is independent of BIP levels [29, 30]. Thus, we aimed to compare the cytotoxic effects of bortezomib and carfilzomib in TNBC cell lines MDA-MB-231 and BT549 as well as in the luminal non-TNBC cell lines MCF-7 and BT474 by exposing the cells to increasing concentrations of PIs for 1 h followed by removal of the drug and subsequent culture in drug-free media for the next 48 h. To validate these data in patients’ primary material, we obtained cancer cells from three patients with TNBC and exposed them to bortezomib and carfilzomib in the same manner. Bortezomib and carfilzomib showed approximately 10x higher cytotoxicity in basal-like TNBC cell lines and primary cells than in luminal BC cell lines (Fig. 2a, b**;** Supplementary Table S3), consistent with a previous report [3]. Next, we aimed to potentiate the cytotoxicity of PI in breast cancer cells by co-treatment with the HIV-PIs nelfinavir and lopinavir, which showed synergistic effect with PI in various types of cancer [31, 32]. Cells were exposed to increasing concentrations of PIs for 1 h followed by continuous exposure to HIV-PI for the next 48 h. The HIV-PIs significantly increased the cytotoxicity of carfilzomib, and to a lesser extent, of bortezomib in TNBC cell lines (Fig. 2c, d). Moreover, nelfinavir and lopinavir showed a strong synergistic effect with carfilzomib, but not with bortezomib in TNBC cells, based on the coefficient of drug interaction (CDI) (Supplementary Figs. S1, S2). Similarly, both HIV-PIs increased the cytotoxicity of the PIs in primary cells. In general, nelfinavir was more effective than lopinavir (Fig. 2e, f), and drugs in combination with bortezomib and carfilzomib showed synergistic effects (Supplementary Figs. S3, S4). At equimolar doses, nelfinavir was more cytotoxic than lopinavir in some cell lines (Fig. 2c–f).

Next, encouraged by the synergistic cytotoxicity of carfilzomib and nelfinavir in primary cells, we tested the combination of carfilzomib and nelfinavir in PDX-derived organoids (PDxOs). In all three models tested, HCI-002, HCI-010, and HCI-023, carfilzomib showed synergistic effects with nelfinavir, as indicated by the synergy score, and for the most effective drug combination, the CDI was calculated (Fig. 2g). Thus, basal-like TNBC is sensitive to proteasome inhibition by carfilzomib, which can be significantly potentiated by nelfinavir or lopinavir.

### Lopinavir in combination with carfilzomib induces UPR, unresolved ER stress, and apoptosis in TNBC

**Fig. 3:**
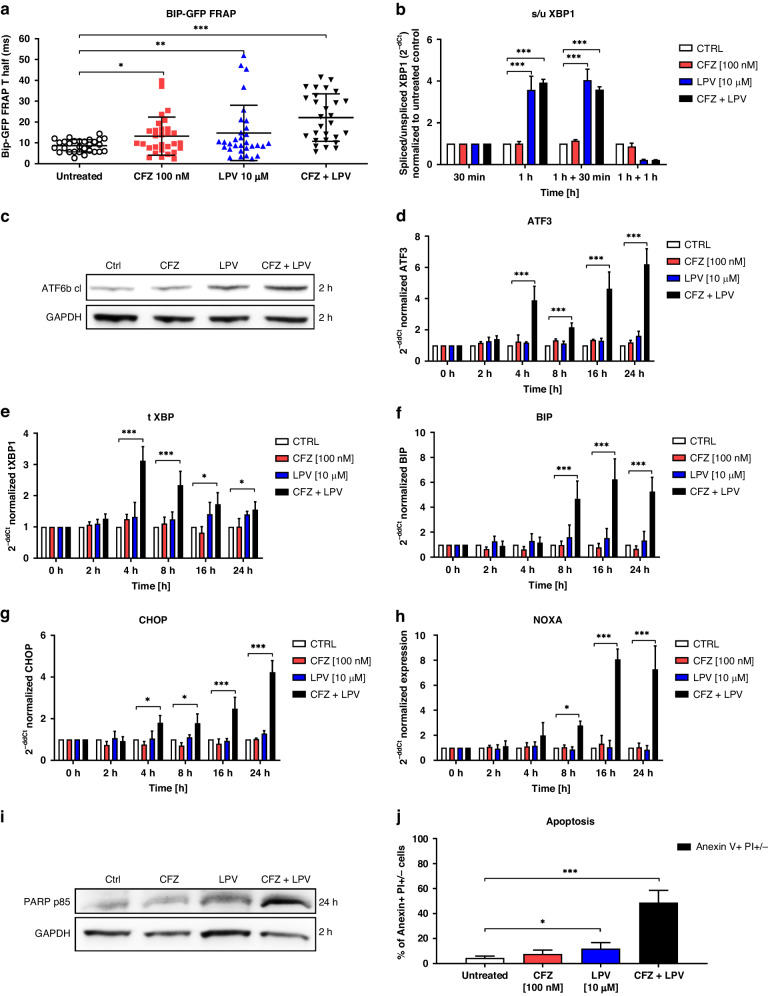
Induction of proteotoxic stress, UPR, and apoptosis in MDA-MB-231 cell line treated with carfilzomib and lopinavir. **a** BiP-GFP FRAP analysis in MDA-MB-231 cells treated with carfilzomib, lopinavir, or their combination. FRAP images were acquired 1 h after the 1 h pulse treatment with carfilzomib or continuous treatment with lopinavir. The data represent the mean ± SD of T half (in ms) recovery of BiP-GFP fluorescence in the individual cells in three independent experiments. Statistical significance was determined with one-way ANOVA with Tukey post-test. * represents *p* < 0.05; ** represents *p* < 0.01; *** represents *p* < 0.001. **b** Induction of spliced XBP1 (sXBP1) presented as a ratio of spliced versus unspliced XBP1 RNA variants normalized to GAPDH and a time-point 30 min prior to the treatment. **c** Representative western blot image of the cleavage of ATF6 protein, represented by a cleaved form of ATF6 and obtained 2 h after the 1 h pulse treatment with carfilzomib or continuous treatment with lopinavir. GAPDH served as an internal loading control. **d** Induction of ATF3 expression normalized to GAPDH and a time-point 0 h after the 1 h pulse treatment with carfilzomib or continuous treatment with lopinavir. **e** Induction of total XBP1 (tXBP1) expression, normalized to GAPDH and a time-point 0 h after the 1 h pulse treatment with carfilzomib or continuous treatment with lopinavir. **f** Induction of BIP expression normalized to GAPDH and a time-point 0 h after the 1 h pulse treatment with carfilzomib or continuous treatment with lopinavir. **g** Induction of CHOP expression normalized to GAPDH and a time-point 0 h after the 1 h pulse treatment with carfilzomib or continuous treatment with lopinavir. **h** Induction of NOXA expression normalized to GAPDH and a time-point 0 h after the 1 h pulse treatment with carfilzomib or continuous treatment with lopinavir. **i** Representative western blot image of the induction of PARP on a protein level, represented by a cleaved form of PARP p85 and obtained 24 h after the 1 h pulse treatment with carfilzomib or continuous treatment with lopinavir. GAPDH served as an internal loading control. **j** Induction of early and late apoptosis represented by Anexin V + and PI− + PI+ positivity, evaluated by flow cytometry 48 h after the 1 h pulse treatment with carfilzomib or continuous treatment with lopinavir. The data represent the mean ± SD from 3 independent experiments. Statistical significance was determined with an unpaired two-sided t-test. * represents *p* < 0.05, *** represents *p* < 0.001. In all qPCR experiments, the data represent the mean ± SD from 3 independent experiments. Statistical significance was determined with one-way ANOVA with Tukey post-test. * represents *p* < 0.05, *** represents *p* < 0.001. ATF3 Activating Transcription Factor 6, ATF6 Activating Transcription Factor 6, BiP-GFP Binding immunoglobulin Protein green-fluorescent protein, BTZ bortezomib, CFZ carfilzomib, CHOP DNA Damage-Inducible Transcript 3, GAPDH Glyceraldehyde-3-Phosphate Dehydrogenase, LPV lopinavir, mero-GFP Mammalian Endoplasmic Reticulum-localized redox-sensitive Green-Fluorescent Protein, NOXA Phorbol-12-Myristate-13-Acetate-Induced Protein 1, PARP Poly(ADP-Ribose) Polymerase 1, XBP1 X-Box Binding Protein 1.

Nelfinavir induces rapid ER stress via lipid-bilayer stress owing to high lipophilicity and predominant membrane localization, which was determined in MDA-MB-231 cells as well [33]. This particular type of ER stress is sensed by IRE1/XBP1 and Activating Transcription Factor 3 (ATF3) pathway activation and induces apoptosis in combination with PIs [34]. We hypothesized that lopinavir likewise induces ER stress, which can potentiate the cytotoxicity of carfilzomib in TNBC. The doses of drugs showing synergistic effects (carfilzomib 100 nM, lopinavir 10 µM) only mildly slowed down BIP kinetics, suggesting the unbinding of BIP from membranes to stabilize unfolded proteins [27]. However, the combination of the drugs showed the most significant effect on BIP kinetics (Fig. 3a). Initially, we observed that lopinavir alone at a given dose induced rapid and quick splicing of XBP1 1 h post-treatment, which was also observed for the combination treatment with carfilzomib, but was not observed with carfilzomib alone (Fig. 3b) and was not accompanied by changes in the level of total XBP1 (Supplementary Fig. S5a). Lopinavir monotherapy induced mild cleavage of ATF6 2 h after treatment, which was potentiated by carfilzomib (Fig. 3c). In agreement with BIP kinetics, none of the drugs at the indicated monotherapy dose further induced pro-apoptotic UPR. In contrast, combination treatment triggered UPR, observed as cleavage of ATF6 2 h post-treatment (Fig. 3c) and transcriptional induction of ATF3, BIP, and total XBP1 4 h post-treatment (Fig. 3d–f), accompanied by mild induction of sXBP1, ATF4, and eIF2a phosphorylation (Supplementary Fig. S5b–d). Next, UPR caused by combination treatment was not resolved during the early hours, eventually triggering apoptosis. This is represented by DNA Damage Inducible Transcript 3 (DDIT3 or CHOP) and Phorbol-12-Myristate-13-Acetate-Induced Protein 1 (NOXA) induction 16 h after treatment (Fig. 3g, h), which is followed by cleavage of Poly(ADP-Ribose) Polymerase (PARP), (Fig. 3i), and phosphatidylserines switch on the plasma membrane 24 h after treatment (Fig. 3j). Thus, a combination of carfilzomib and lopinavir induced strong terminal UPR, leading to apoptosis.

### Spliced XBP1 is a surrogate marker for response to proteasome inhibitors and is not a direct target of HIV-protease inhibitors

**Fig. 4:**
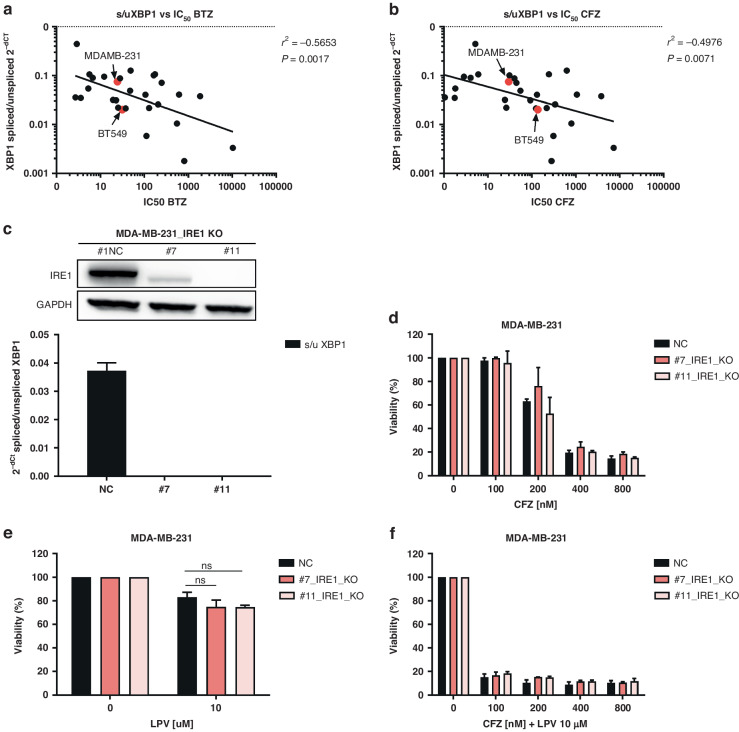
Dissection of the role of sXBP1 in monotherapy with carfilzomib and lopinavir or in the drug combination in MDA-MB-231. **a** Correlation between spliced XBP1 expression level, represented as a ratio of spliced / unspliced XBP1 and normalized to GAPDH, and the sensitivity to bortezomib, represented by the IC_50_ values. **b** Correlation between spliced XBP1 expression level, represented as a ratio of spliced/unspliced XBP1 and normalized to GAPDH, and the sensitivity to carfilzomib, represented by the IC_50_ values. In both **a** and **b**, TNBC are marked in red. The data represent the mean of two independent experiments. **c** Representative western blot image of IRE1α knock-out in control or in two single-cell derived clones #7 and #11 of MDA-MB-231 cell line (upper part), leading to a functional decrease of sXBP1 evaluated by qPCR, assessed as a ratio of spliced vs unspliced XBP1 and normalized to GAPDH, which served as a housekeeping gene (bottom part). **d** Cytotoxicity of carfilzomib in MDA-MB-231 cells with a normal level of IRE1α (NC) or in single-cell derived clones (#7 and #11) with knocked-out IRE1α. Viability was assessed after 1 h pulse treatment and continuous 48 h incubation in drug-free medium. The data represent the mean ± SD from 3 independent experiments. Statistical significance was determined with an unpaired *t*-test. **e** Cytotoxicity of lopinavir in MDA-MB-231 cells with a normal level of IRE1α (NC) or in single-cell derived clones (#7 and #11) with knocked-out IRE1α. Viability was assessed after 48 h continuous treatment. The data represent the mean ± SD from 3 independent experiments. Statistical significance was determined with unpaired *t*-test. **f** Cytotoxicity of carfilzomib and lopinavir combination in MDA-MB-231 cells with a normal level of IRE1α (NC) or in single-cell derived clones (#7 and #11) with knocked-out IRE1α. Viability was assessed after 1 h pulse treatment with carfilzomib and continuous 48 h treatment with lopinavir. The data represent the mean ± SD from 3 independent experiments. Statistical significance was determined with an unpaired t-test. BTZ bortezomib, CFZ carfilzomib, GAPDH Glyceraldehyde-3-Phosphate Dehydrogenase, IRE1α Inositol-Requiring Enzyme 1, LPV lopinavir, XBP1 X-Box-Binding Protein 1.

Levels of sXBP1 are predictors of sensitivity to bortezomib in MM [29, 30]. Using 25 different cancer cell lines (specified in Supplementary data), we showed that the levels of sXBP1 significantly correlate with bortezomib and carfilzomib sensitivity not only in MM, but also in multiple other hematologic and solid cancer cell lines, including TNBC (Fig. 4a, b and Supplementary Table S4).

Extensive splicing of XBP1 by lopinavir in MDA-MB-231 cells led us to further dissect the role of sXBP1 in the cytotoxicity of lopinavir and carfilzomib. Using the CRISPR/Cas9 approach, we knocked out IRE1α in MDA-MB-231 and MCF-7 cell lines (Fig. 4c and Supplementary Fig. S6a). IRE1α knockout significantly depleted the spliced form of XBP1 in both cell lines, irrespective of TNBC status (Fig. 4c and S2a), but had no significant effect on the sensitivity of cells to lopinavir or carfilzomib, either individually or in combination (Fig. 4d–f for MDA-MB-231 and Supplementary Fig. S6b–d for MCF-7). Thus, although high sXBP1 is a marker of basal ER stress and pro-survival UPR activation in TNBC, manipulation of sXBP1 levels did not change the sensitivity to PIs, suggesting that sXBP1 is a surrogate marker of response to PIs. The data also suggest that the synergy between HIV-PIs and carfilzomib is not mediated solely by XBP1 induction.

### Lopinavir potentiates the cytotoxicity of the β5 + β2-pattern of proteasome inhibition induced by carfilzomib

The HIV-PIs nelfinavir and lopinavir potentiate the activity of PIs in MM and other hematological malignancies [11, 33, 35] via modulation of proteasome activity or assembly, inhibition of efflux transporters that transport PI outside of the cells, or changing lipid membrane fluidity [11, 31, 35, 36]. While nelfinavir, as being more lipophilic drug, induces lipid membrane rigidification, lopinavir is a more potent modulator of ABCB1 efflux pump activity, allowing for more effective proteasome inhibition provided by PIs [11, 33]. To determine whether lopinavir potentiates the efficacy of carfilzomib in TNBC by modulating the proteasome itself or by increasing the intracellular availability of carfilzomib, we studied the effect of both drugs and their combination directly on the inhibition of catalytically active proteasome subunits by ABP, as well as on functional proteasome inhibition and corresponding cell viability. For this purpose, we established an MDA-MB-231 cell line stably expressing Ub^G76V^-GFP, where GFP is directly targeted for proteasomal degradation and its intensity is directly proportional to the degree of proteasome inhibition.

**Fig. 5:**
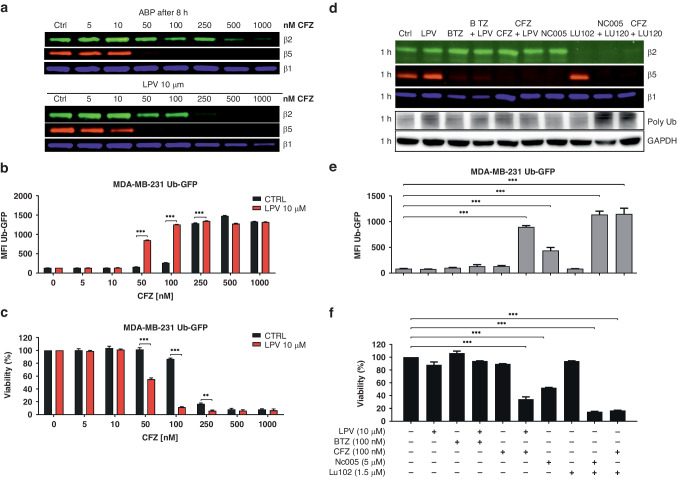
Effect of the drugs and their combination on proteasome activity in MDA-MB-231 cells stably expressing Ub^G76V^-GFP. **a** Representative gel image of residual activity of the proteasome β2, β5, and β1 subunits, visualized by ABP labelling 8 h after the treatment. MDA-MB-231_Ub^G76V^GFP cells were treated with carfilzomib alone for 1 h and subsequently placed into drug-free medium or into medium containing 10 µM lopinavir for 8 h. **b** Median fluorescence intensity (MFI) of Ub^G76V^-GFP, corresponding to functional proteasome inhibition in cells treated with carfilzomib alone or co-treated with lopinavir for 8 h. The data represent the mean ± SD from 3 independent experiments. Statistical significance was determined with two-way ANOVA and Sidak’s post-test, *** represents *p* < 0.001. **c** Viability corresponding to **a** and **b**. The cells were treated with carfilzomib for 1 h and subsequently placed into a drug-free medium or into the medium with lopinavir for 48 h. The data represent the mean ± SD from 3 independent experiments. Statistical significance was determined with two-way ANOVA and Sidak’s post-test, ** represents *p* < 0.01; *** represents *p* < 0.001. **d** Representative gel image of residual activity of the proteasome β2, β5, and β1 subunits, visualized by ABP labelling and corresponding poly-Ub accumulation 1 h after the treatment. MDA-MB-231_Ub^G76V^GFP cells were treated for 1 h with 10 µM lopinavir, 100 nM bortezomib, or 100 nM carfilzomib in monotherapy or in combination, as well as with β5 specific inhibitor NC005, β2 specific inhibitor LU102 or their combination. **e** MFI of Ub^G76V^-GFP, representing functional proteasome inhibition 8 h after the treatment. The cells were treated with PIs, as shown in **d** for 1 h and subsequently placed into a drug-free medium or into the medium with lopinavir for 8 h. The data represent the mean ± SD from 3 independent experiments. Statistical significance was determined with one-way ANOVA with Dunnet post-test, *** represents *p* < 0.001. **f** Viability corresponding to **d** and **e**. The cells were treated with PIs for 1 h and subsequently placed into a drug-free medium or into a medium containing lopinavir for 48 h. The data represent the mean ± SD from 3 independent experiments. Statistical significance was determined with one-way ANOVA with Dunnet post-test, *** represents *p* < 0.001. BTZ bortezomib, CFZ carfilzomib, GAPDH Glyceraldehyde-3-Phosphate Dehydrogenase, LPV lopinavir, MFI median fluorescence intensity, Ub^G76V^-GFP mutated uncleavable ubiquitin moiety-Green Fluorescent Protein.

ABP labelling after 1 h of pulse treatment with carfilzomib alone showed inhibition of the β5 subunit already at 10 nM drug concentration and complete β5 inhibition at 50 nM drug concentration. One-hour pulse treatment with 10 µM lopinavir monotherapy showed no inhibition of the catalytic activity of any of the proteasome β subunits, whereas in the co-treatment setup, it increased the potency of carfilzomib to inhibit the β5 subunit already at 5 nM (Fig. 5a). Subsequent evaluation of proteasome activity 7 h after the 1 h pulse treatment with carfilzomib showed complete restoration of β5 activity at 10 nM; however, the combination of pulse treatment of carfilzomib with continuous treatment of lopinavir inhibited β5 activity at 10 nM concentration, decreased β2 activity at 50 nM dose and β1 activity at 250 nM. These data suggest that lopinavir allows for increased intracellular concentrations of carfilzomib, which more potently inhibits proteasomes at the β5 and β2 sites, as well as β1, at very high doses. At the same time, this shows that upon co-treatment, the recovery of proteasome activity is slower. Functional inhibition of proteasome, represented by GFP accumulation, confirmed that carfilzomib alone functionally inhibits the proteasome at 100 nM concentration with full inhibition at 250 nM; however, in combination with lopinavir, carfilzomib induced functional proteasome inhibition already at 50 nM drug concentration with full inhibition at 100 nM drug concentration (Fig. 5b). Higher functional inhibition of proteasome directly corresponded to increased cytotoxicity measured 48 h post treatment (Fig. 5c). To further assess whether the β5 and β2 co-inhibition of the proteasome leads to the most effective functional proteasome inhibition and cytotoxicity in TNBC, as we have previously shown in MM [8], we analyzed the inhibition profile of proteasome β subunits by ABP after 1 h exposure to proteasome-inhibiting approved drugs or experimental subunit-selective PIs (bortezomib, carfilzomib, β5 specific inhibitor NC005, β2 specific inhibitor LU102, and their combination), and the corresponding accumulation of GFP and cytotoxicity (Fig. 5d–f). Although 100 nM bortezomib completely inhibited β5 and β1 subunits, it did not cause strong functional proteasome inhibition or cytotoxicity, and co-treatment with lopinavir did not increase the functional proteasome inhibition and cytotoxicity. In contrast, only minor inhibition of the β2 subunit, together with total β5 inhibition, achieved by the combination of carfilzomib and lopinavir, induced more effective functional proteasome inhibition and cytotoxicity. Importantly, strong functional proteasome inhibition associated with high cytotoxicity was recapitulated by NC005 + LU102 treatment, carfilzomib + LU102 treatment (Fig. 5d–f) or bortezomib co-treatment with LU102 and lopinavir (Supplementary Fig. S7). We further showed that the unique β5/β2 proteasome inhibition profile reached by high-dose carfilzomib causes the strongest functional inhibition of the proteasome and cytotoxicity in TNBC, in comparison to boronate-based approved PI bortezomib and ixazomib, which inhibit β5/β1 proteasome subunits (Supplementary Fig. S8a–c). In short, the inhibition of β5/β2 subunits provided by the clinically approved PI carfilzomib is the most effective in inducing cytotoxicity in TNBC, and lopinavir can increase the intracellular availability of carfilzomib to achieve more effective proteasome inhibition.

### Inhibition of ABCB1, but not ABCG2 activity by lopinavir increases the cytotoxicity of carfilzomib

**Fig. 6:**
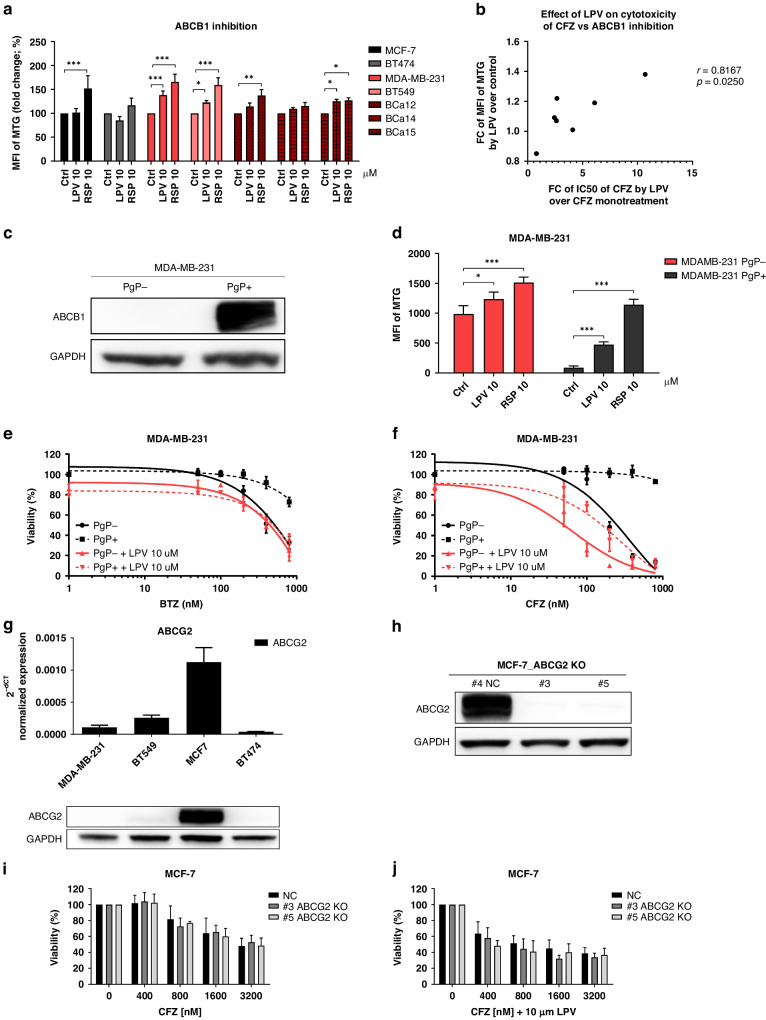
Involvement of multi-drug resistance transporters in CFZ sensitivity. **a** Functional inhibition of ABCB1 (PgP) multi-drug transporters by lopinavir and reserpine in 4 studied cell lines and patient-derived primary cells. The data represent the mean MFI of MTG ± SD from 3 independent experiments for cell lines and the single value for patient-derived primary samples. Statistical significance was determined with unpaired *t*-test, ** represents *p* < 0.01; *** represents *p* < 0.001. **b** Correlation between fold change of MFI of MTG by lopinavir and fold change of IC_50_ of carfilzomib by lopinavir. The data represent the correlation between the mean values from three independent experiments from cell lines and single values from patient-derived primary cells. The *p*-value and correlation coefficient (r) were determined by Pearson correlation. **c** Representative western blot image of ABCB1 level in MDA-MB-231 cells stably transduced with ABCB1 protein (PgP+). GAPDH serves as a protein loading control. **d** Functional inhibition of ABCB1 by lopinavir and reserpine in PgP+ and PgP- MDA-MB-231 cells. The data represent the mean MFI of MTG ± SD from 3 independent experiments. Statistical significance was determined with unpaired t-test, ** represents *p* < 0.01; *** represents *p* < 0.001. Dose-response curves of MDA-MB-231 cells without PgP (PgP-) or with introduced PgP (PgP + ) to bortezomib (**e**) and carfilzomib (**f**) in monotherapy or in combination with lopinavir. The cells were treated with PIs for 1 h and subsequently placed into a drug-free medium or into the medium with lopinavir for 48 h. The data represent the mean ± SD from 3 independent experiments. **g** Estimation of ABCG2 in 4 studied cell lines on the mRNA level by qPCR (upper part) and on the protein level (lower part) by western blot. In both experiments, the levels were normalized to GAPDH, which served as a control. **h** Representative western blot image of ABCG2 knock-out in control or in two single-cell derived clones #3 and #5 of MCF-7 cell line. **i** Cytotoxicity of carfilzomib in MCF-7 cells with a normal level of ABCG2 (NC) or in single-cell derived clones (#3 and #5) with knocked-out ABCG2. Viability was assessed after 1 h pulse treatment with carfilzomib and continuous 48 h incubation in a drug-free medium. The data represent the mean ± SD from 3 independent experiments. Statistical significance was determined with an unpaired t-test. **j** Cytotoxicity of carfilzomib and lopinavir combination in MCF-7 cells with a normal level of ABCG2 (NC) or in single-cell derived clones (#3 and #5) with knocked-out ABCG2. Viability was assessed after 1 h pulse treatment with carfilzomib and continuous 48 h treatment with lopinavir. The data represent the mean ± SD from 3 independent experiments. Statistical significance was determined with an unpaired *t*-test. ABCB1 ATP Binding Cassette Subfamily B Member 1, ABCG2 ATP Binding Cassette Subfamily G Member 2, BTZ bortezomib, CFZ carfilzomib, GAPDH Glyceraldehyde-3-Phosphate Dehydrogenase, LPV lopinavir, MFI median fluorescence intensity, MTG MitoTracker Green FM, PgP P-glycoprotein (ABCB1).

The major factors preventing carfilzomib efficacy in tumor cells are its low tissue penetration and high affinity for ABCB1 transporters [8, 11, 14]. Thus, we aimed to determine the expression and activity of ABCB1 in the four studied cell lines and patient’s primary samples. ABCB1 expression was low but detectable in all cell lines studied. A functional efflux assay using Mitotracker Green FM (MTG), a known substrate of ABCB1 [37], showed that it retained activity that could be inhibited by lopinavir in TNBC, but not in the other two non-TNBC cell lines (Fig. 6a). As a positive control, we used reserpine, a known ABCB1 inhibitor that shows more effective retention of MTG across the cells, putatively due to the different molecular mechanisms of ABCB1 modulation (Fig. 6a). Importantly, we observed a significant correlation (Pearson correlation coefficient *r* = 0.8167, *p* < 0.05) between the fold change in MTG efflux inhibition and the fold change in increased cytotoxicity to carfilzomib caused by lopinavir (Fig. 6b, Supplementary Table S4). Next, we observed that lopinavir mildly inhibited ABCB1 efflux in the patient’s primary cells. For carfilzomib is a stronger ABCB1 substrate than bortezomib, the presence of ABCB1 likely explains the slightly increased cytotoxicity of carfilzomib and lopinavir over bortezomib and lopinavir combination in these cells.

To confirm that lopinavir sensitized TNBC cells to carfilzomib via ABCB1 inhibition, we overexpressed ABCB1 (PgP) in MDA-MB-231 cells using a retroviral construct. The cells showed high PgP positivity at the protein and functional levels, as they strongly efflux MTG out of the cells (Fig. 6c, d). Lopinavir (10 µM) inhibited the efflux of MTG in these cells, whereas the efflux was more potently inhibited by 10 µM reserpine (Fig. 6d). We exposed strongly and mildly positive PgP cells to bortezomib and carfilzomib alone or in combination with 10 µM lopinavir. Strong PgP positivity increased resistance of the cells to carfilzomib and, to a lesser extent, bortezomib. In both settings, co-treatment with 10 µM lopinavir reversed the cytotoxicity to the level of PgP mildly positive cells (Fig. 6e, f). Similar results were also observed for 10 µM nelfinavir (Supplementary Fig. S8a, b).

Breast cancer cells predominantly overexpress other types of pumps such as the breast cancer-related protein ABCG2. Therefore, we assessed its role in carfilzomib efflux and inhibition by lopinavir. Of the four cell lines used in this study, MCF-7 cells overexpressed ABCG2 (Fig. 6g). We knocked out ABCG2 in a highly positive MCF-7 cell line and in a poorly positive MDA-MB-231 cell line, and exposed the cells to carfilzomib and lopinavir individually or in combination (Fig. 6h and Supplementary Fig. S8c). ABCG2 KO did not significantly sensitize the cells to carfilzomib (Fig. 6i and Supplementary Fig. S8d). Likewise, it did not significantly affect the cytotoxicity of combination treatment in MCF-7 cells, but mildly decreased the cytotoxicity in MDA-MB-231 cells (Fig. 6j and Supplementary Fig. S8e). These data show that ABCB1 is the main limiting drug transporter of carfilzomib, which is present in TNBC cell lines and primary cells.

## Discussion

Proteasome inhibitors are the backbone of treatment for MM and mantle cell lymphoma. Bortezomib has been tested in clinical trials in solid tumors, but failed to show clinical benefit in breast cancer, possibly in part due to poor penetration into solid tumors and partly because only a subset of patients shows proteasome dependency, predicting their vulnerability to proteasome inhibition. Carfilzomib could be a therapeutic option for this subset of patients if relevant concentrations of carfilzomib are reached. Our results demonstrate that targeting the proteasome in TNBC cells, in particular with carfilzomib in combination with the anti-HIV drugs nelfinavir or lopinavir, has a synergistic cytotoxic effect not only in cell lines, but also in TNBC patient-derived cells and organoids caused by i) sensitization of the cells towards carfilzomib, leading to superior functional inhibition of the proteasome, and ii) induction of ER stress, leading to activation of apoptosis.

Next-generation PIs have been developed to specifically inhibit the proteasome β5 subunit, which is the rate-limiting protease in MM, non-Hodgkin lymphoma, and leukemia cells [38]. However, in non-hematologic tumors, more effective inhibition of the proteasome is needed to induce cytotoxic effects, as the β5 site is not rate-limiting for many proteins, and significant inhibition of protein degradation is obtained only by the inhibition of the β5 sites, and either the β2 or β1 sites, while the relative importance of the three active sites depends on the proteins being degraded [39]. By using specific activity-based proteasome probes, we have previously shown that higher, but clinically relevant concentrations of bortezomib inhibit β5 and co-inhibit the β1 subunit, while higher concentrations of carfilzomib co-inhibit the β2 subunit [8, 9]. Importantly, continuous β5 inhibition leads to extensive upregulation of β2 activity, observed upon bortezomib treatment or in bortezomib-adapted MM cells [40]. Likewise, selective co-inhibition of β2 can overcome resistance to bortezomib and increase its cytotoxicity [41]. Here, we show that TNBC resembles MM in dependency on the proteasome and specifically in the activity of the β5 and β2 subunits, which can be inhibited by clinically relevant doses of carfilzomib [9]. Furthermore, the cells are more sensitive to carfilzomib than to bortezomib in combination with lopinavir or nelfinavir, as only carfilzomib is able to prevent β2 upregulation or decrease β2 activity in the presence of the inhibited β5 proteasome subunit. Since carfilzomib is a strong substrate of the ABC-type transporter ABCB1 and lopinavir is an effective inhibitor of ABCB1 [11], the combination of carfilzomib and lopinavir allows for stronger functional proteasome inhibition in the presence of ABCB1. Our data further showed that carfilzomib is a poor substrate of ABCG2 and that lopinavir does not increase the intracellular availability of carfilzomib via ABCG2 inhibition.

HIV-PIs, such as saquinavir, nelfinavir, and lopinavir, have been described as potent inducers of ER stress, which at least for nelfinavir can be explained by its high lipophilicity and strong binding to lipid-rich membranes [33]. HIV-PIs cause upregulation of cytosolic and ER-resident heat shock proteins and induce apoptosis in cancer cells associated with caspase activation and induction of the pro-apoptotic transcription factor CHOP [32, 35]. Furthermore, lopinavir has been shown to induce strong ER stress not via proteasome inhibition, but rather via reactive oxygen species-dependent Mitogen-Activated Protein Kinase 8 (JNK) activation [42] and has been previously described to induce ER stress in breast cancer cells, either triple-negative or non-triple-negative [43, 44]. Our data show that lopinavir induced mild impairment of BIP mobility in TNBC, but rapid splicing of XBP1, which is likely not associated in this early stage with ER stress caused by the accumulation of unfolded proteins. However, in combination with carfilzomib, BIP mobility is significantly impaired, suggesting the accumulation of unfolded proteins that induce broad ER stress. Following, ER stress is not resolved, triggering the expression of BIP and pro-apoptotic regulators CHOP and NOXA, subsequently leading to apoptosis. Based on previous evidence [13], we observed that TNBC cells have higher basal levels of pro-survival UPR activation as represented by increased sXBP1 and BIP. Given that TNBC cells are linked to heightened aggressiveness and poor prognosis because of i) a high rate of mutations in oncogenes and tumor suppressors, such as Tumor Protein P53 (TP53) and Neurofibromin 1 (NF1) [45]; ii) activation of pathways associated with metastasis, such as extracellular matrix-receptor interaction, cell adhesion, and angiogenesis [45] and iii) extensive metabolic changes, including dependency on Glutathione S-Transferase Pi 1 (GSTP1) as a regulator of energy and glycolytic and lipid metabolism [46], they likely produce excessive amounts of mutated proteins that are folded in an error-prone manner and thus are more dependent on ER-associated degradation. In contrast, non-TNBC cells show accumulation of PDI, which allows for effective protein disulfide bond pairing, possibly ensuring a lower dependency on protein degradation by the proteasome. Consistent with this, luminal-like cell lines, which are mostly non-TNBC, have increased levels of proteins in the pathways associated with proliferation (cell cycle, growth factor signaling, and DNA damage repair mechanisms) and metabolism compared to TNBC [45], suggesting a higher proliferation rate accompanied by upregulation of redox homeostasis.

Our data suggest that the features of TNBC, especially the strong dependence on functional proteasome, resemble those of MM and make TNBC candidates for carfilzomib-based therapy in combination with HIV-protease inhibitors. Accordingly, recent data suggest PIs as promising compounds to be used in combination therapy for TNBC [47]. A therapeutic combination of PIs with HIV-protease inhibitors showed promising results in relapsed myeloma patients. The combination of nelfinavir and bortezomib triggered UPR directly in patients and showed an overall response rate of 65% in a Phase II trial of relapsed/refractory MM patients [48, 49]. At the same time, lopinavir-ritonavir addition to carfilzomib therapy induced a partial response in half of the cases of carfilzomib-refractory MM and was well tolerated [50]. Thus, the therapy combination has been shown to be safe and tolerable and its efficacy in patients with TNBC remains to be elucidated. We acknowledge, that carfilzomib has shown limited efficacy in solid tumor therapy due to the short half-life and poor tumor distribution. To overcome this limitation, infusion with a dose over 36 mg/m2 in patients, leading to stronger functional proteasome inhibition, or a modulated formulation of the drug may overcome these obstacles, as has been shown [9, 51].

Importantly, tumor cells often express another type of proteasome, the immunoproteasome, which is typically found in cells of hematopoietic origin. Recent data show that TNBC is enriched in immunoproteasomes, with higher expression of the immunoproteasome β5i subunit correlating with higher densities of tumor-infiltrating lymphocytes [52]. Since PIs bortezomib and carfilzomib target both types of proteasomes, proteasome inhibition in tumor-infiltrating lymphocytes may be actually counterproductive, suggesting decreased tumor immunogenicity. Thus, further studies are needed to elucidate the proper therapeutic strategy and combinations with PIs.

In conclusion, we show that HIV-protease inhibitors, increase the cellular availability of carfilzomib in TNBC cell lines, TNBC patient-derived tumor cells ex vivo, and TNBC-derived organoids, leading to stronger functional proteasome inhibition and UPR activation in TNBC cells, terminally triggering cellular apoptosis. We believe, that carfilzomib in combination with nelfinavir or lopinavir-ritonavir could serve as a viable treatment option for patients in resource-limited settings who have already undergone all currently accessible therapies.

### Supplementary information

Supplemental data

## Data Availability

The datasets generated during this study are available from the corresponding authors upon reasonable request.
