# Supplementary material for: HIV-protease inhibitors potentiate the activity of carfilzomib in triple-negative breast cancer
Source: Br J Cancer. 2024 Jul 5;131(5):918–30. doi: 10.1038/s41416-024-02774-9 (PMC11368961; doi:10.1038/s41416-024-02774-9)
Supplement: Supplementary file 1 — Supplemental data [file 41416_2024_2774_MOESM1_ESM.docx]

**SUPPLEMENTARY DATA**

**HIV-protease inhibitors potentiate the activity of carfilzomib in triple-negative breast cancer**

Andrej Besse, Lenka Sedlarikova, Lorina Buechler, Marianne Kraus, Chieh-Hsiang Yang, Nicol Strakova, Karel Soucek, Jiri Navratil, Marek Svoboda, Alana L. Welm, Markus Joerger, Christoph Driessen, Lenka Besse

**SUPPLEMNETARY METHODS**

**Viability assay**

The cell viability assay was performed by seeding 5000 cells/well in 100 μl of growth medium in 96-well plates. After 24 h, drugs or their combination diluted in 100 μl of the medium were added to each well for 1 h pulse and then the medium was replaced with drug-free medium or medium containing lopinavir/nelfinavir for a subsequent 48 h treatment. The viability of the cell lines was determined by MTS tetrazolium compound using CellTiter 96® AQueous One Solution according to the manufacturer’s recommendations (Promega, Madison, WI, USA; Promega). The viability of primary cells was determined after 48 h of treatment using the CellTiter-Glo® Luminescent Cell Viability Assay according to the manufacturer’s recommendations (Promega). IC_50_ values were determined by GraphPad Prism software using linear regression function.

The coefficient of drug interaction (CDI) was calculated as follows: CDI = AB/(A×B). According to the viability of each group, AB is the ratio of the viability of the combination group to that of the control group and A or B is the ratio of the viability of the single-agent group to that of the control group.

**PDxO culture and three-dimensional synergy drug testing**

In brief, PDxOs (HCI-002, HCI-010, and HCI-023) were maintained in 6-well plates (Genesee Scientific, El Cajon, CA, USA) and cultured in 200-μl Matrigel (Corning, Corning, NY, USA) domes in Advanced DMEM/F12 (Thermo Fisher, Waltham, MA, USA) supplemented with 5% FBS, 10 mM HEPES (Thermo Fisher, Waltham, MA, USA), 1× Glutamax (Thermo Fisher, Waltham, MA, USA), 1 μg/ml hydrocortisone (Sigma-Aldrich, Burlington, MA, USA), 50 μg/ml gentamicin (Genesee Scientific, El Cajon, CA, USA), 10 ng/ml hEGF (Sigma-Aldrich, Burlington, MA, USA), and 10 μM Y-27632 (Selleck Chemicals, Houston, TX, USA). Mature organoids were collected from culture using dispase (Fisher Scientific, Waltham, MA, USA) treatment (40U per well) at 37°C for 25 minutes. Approximately 40-50 matured organoids (~70 μm in diameter) were seeded per well in 384-well tissue culture plates (PerkinElmer, Waltham, MA, USA), each comprising a solidified 10-μl Matrigel base layer and 30 μl of PDxO culture medium. Plates were then incubated at 37°C and 5% CO2 overnight to allow the organoids to settle onto the Matrigel base layer. A separate 1 ml deep 96-well drug plate was prepared with an eight-point (nelfinavir) x eleven-point (carfilzomib) serial dilution, and 30 μl of each condition, in technical quadruplicate, was transferred to the seeded 384-well plates. The dosed PDxO plates were covered with Breathe-Easy seals (USA Scientific, Ocala, FL, USA) and incubated for 144 hrs at 37°C and 5% CO2. After incubation, the seals were removed, and 15 μl of CellTiterGlo 3D (Promega, Madison, WI, USA) was added to each well. The plates were then incubated on a plate shaker (Benchmark Scientific, Sayreville, NJ, USA) at 500 rpm for 20 minutes and assayed with the EnVision XCite plate reader (PerkinElmer, Waltham, MA, USA). Raw luminescent values from each condition were divided by the values from the untreated wells to obtain viability. Synergy maps and scores were generated using SynergyFinder+ (3,4).

**Generation of MDA-MB-231 cell line expressing Ub^G76V^-GFP**

A lentiviral vector encoding unstable GFP (Ub^G76V-^GFP, containing a mutated uncleavable ubiquitin moiety, obtained from Nico Dantuma; Addgene plasmid #11941 (1)) was generated by cloning the sequences encoding Ub^G76V-^GFP into the lentiviral transfer vector pLenti-Puro (Addgene #39481) using SpeI/EcoRI (New England Biolabs, Ipswitch, MA, USA) (1). Lentivirus was produced using a lentiviral transfer vector and packaging plasmids psPAX2 and pMD2.G (a gift from Trono’s lab; Addgene plasmids #12260 and #12259) in HEK293T-LentiX cells (Takara Bio/Clontech, Kusatsu, Japan; Takara Bio). Stably transduced MDA-MB-231 cells were selected using puromycin (Merck/Sigma-Aldrich, Buchs, Switzerland; Merck). Subsequently, cells were subcloned, and the single-cell-derived cell line with the most optimal accumulation of fluorescence upon reversible proteasome inhibition was chosen for further analyses.

**Generation of ABCB1 expressing MDA-MB-231 cell line**

MDA-MB-231 cells were transduced with the retroviral vector pHaMDRwt (a gift from Gottesman’s lab; Addgene plasmid #10957). Retroviruses were produced by packaging plasmids pUMVC and pMD2.G (Gifts from Weinberg’s and Trono’s lab; Addgene plasmids #8449 and #12259) in HEK293T-LentiX cells (Takara Bio). Stably transduced cells were selected with puromycin (Merck).

**Generation of MERO-GFP expressing cell lines**

The MERO-GFP construct (mammalian endoplasmic reticulum-localized redox-sensitive green fluorescent protein, a gift from Prof. Urano, Department of Medicine, Division of Endocrinology, Metabolism, and Lipid Research, Washington, USA) is an ER-specific redox sensor equipped with the signal sequence of mouse BiP and the mammalian ER retention signal KDEL to the N-terminus and C-terminus of the redox-sensitive GFP, respectively, which display distinct excitation spectra in the fully oxidized and reduced states, with maxima at 394 nm and 473 nm, respectively (2, 3). MDA-MB-231, BT549, MCF-7, and BT474 cells were equipped with the construct by lentivirus transduction. Briefly, lentiviruses were produced by packaging plasmids pMD2.G and psPAX2 (a gift from Trono’s lab; Addgene plasmids #12259 and #12260) and the MERO-GFP transfer plasmid in the HEK-293-LentiX cell line (Takara Bio). After transduction, MERO-GFP-positive cells were selected using puromycin (Merck).

**Generation of BIP-mGFP expressing cells**

BiP-mGFP (obtained from Erik Snapp; Addgene plasmid #62231) encodes a hamster BiP/GRP78/HSPA5 fused to monomeric EGFP, which is useful for studying BiP mobility and trafficking in live cells (4). MDA-MB-231, BT549, MCF-7 and BT474 cells were equipped with the construct via electroporation. Cells with stable expression of the construct were selected using gentamicin (Merck), after which the cells were subcloned, and the clone with the most optimal fluorescence was chosen for further analyses.

# CRISPR/Cas9 knock-out

The two-vector CRISPR/Cas9 system was introduced into MDA-MB-231 and MCF-7 cells by lentiviral infection (5, 6). Lentiviruses were produced using the packaging plasmids psPAX2 and pMD2.G (a gift from Trono’s lab; Addgene plasmids #12260 and #12259) and transfer plasmids lentiCas9-Blast or lentiGuide-Puro (a gift from Zhang’s lab; Addgene plasmids #52962 and #52963) in HEK293T-LentiX cells (Takara Bio). This two-vector system allows the delivery of Cas9 and sgRNA on separate vectors with distinct antibiotic selections. After Cas9 infection, cells were selected using Blasticidine S (Merck). Cells with stably introduced Cas9 were infected with particles containing sgRNA targeting IRE1α, exon 4 (sgRNA sequence was designed using the online tool crispr.mit.edu; sgRNA for IRE1: TTCTCCCAGATCCTAATGA), and ABCG2 exon 2 (sgRNA sequence was designed using the online tool crispr.mit.edu; sgRNA for ABCG2: GGTCATTGGAAGCTGTCGCG) and selected by puromycin (Merck). Cells were subcloned, clones screened for the mutation using the T7E1 assay (New England Biolabs, Ipswitch, MA, USA), and tested for protein knockdown by western blotting. Sanger sequencing was performed to confirm the presence of a mutation in the desired part of the genome using the forward primer for IRE1α/exon 4 (5’-TGCGTGGCTTTGGTATGTTG-3’) and ABCG2/exon 2 (5’-GTTTATGCATTCCAAGTTGTGC-3’). Two clones with decreased levels of the desired protein were used for further analysis and compared to non-mutated clones.

**Analysis of correlation between the cytotoxicity of bortezomib and carfilzomib and sXBP1**

The following cell lines were used in the analysis: multiple myeloma (AMO-1, AMO-CFZ, AMO-CFZ, RPMI-8226, RPMI-BTZ, MM1S, MM1R, ARH77, ARH-BTZ, L363), breast cancer (MDA-MB-231, BT-474, BT-549, MCF-7), diffuse large B-cell lymphoma (HBL), pancreatic carcinoma (Panc-1, MiaPaCa, Colo357), neuroblastoma (SK-N-AS), osteosarcoma (U2OS), fibrosarcoma (HT1080), clear cell carcinoma (Caki-1), lung carcinoma (A549), glioblastoma (LN229, U251) hepatocyte carcinoma (Hep2G), lung carcinoma (H460), cervix adenocarcinoma (HeLa). Twelve hours prior to the experiments, the suspension and adherent cells were seeded in a flat-bottom 96-well plate. Subsequently, cells were treated with increasing doses of bortezomib and carfilzomib (0 – 800 nM) for 48 h. The mean IC_50_ values for each drug and cell line were derived from the dose-response curves of two independent experiments and are presented in Supplementary Table S4. The ratio of spliced to unspliced XBP1 was determined by qPCR, using the primers specified in Supplementary Table S2. Correlation analysis was performed using Pearson´s correlation coefficient.

**SUPPLEMENTARY TABLES**

**Supplementary Table S1:** Basic characteristics of females diagnosed with triple-negative breast cancer and their tumors.

| **Basic characteristics** | **BCa12** | **BCa14** | **BCa15** |
| --- | --- | --- | --- |
| **Age at the time of diagnosis** | 37 | 84 | 66 |
| **Grade** | 3 | 3 | 3 |
| **Histology** | NST Ca | metaplastic (squamous) Ca | invasive NST Ca |
| **pT** | c2 | 0 | 0 |
| **pN** | 0 | 0 | 0 |
| **M** | 0 | 0 | 0 |
| **ER positivity (%)** | 5 | 0 | 0 |
| **PR positivity (%)** | 0 | 0 | 0 |
| **Her2 positivity (%)** | 0 | 0 | 0 |
| **Ki-67 positivity (%)** | 95 | 92 | 75 |

TNM classification: T: size or direct extent of the primary tumor; N: degree of spread to regional lymph nodes; M: presence of distant metastasis.

ER: Estrogen receptor; Her2: Human epidermal growth factor receptor 2; Ki-67: Marker Of Proliferation Ki-67; PR: Progesterone receptor.

**Supplementary Table S2:** Sequences of primers used for qPCR analysis.

| **SYBR Green** | | |
| --- | --- | --- |
| **PRIMER** | **Forward** | **Reverse** |
| **XBP1 spliced**^2^ | 5′-CTGAGTCCGAATCAGGTGCAG-3′ | 5′-ATCCATGGGGAGATGTTCTGG-3′ |
| **XBP1 unspliced**^2^ | 5′-CAGCACTCAGACTACGTGCA-3′ | 5′-ATCCATGGGGAGATGTTCTGG-3′ |
| **BIP**^2^ | 5′-TGTTCAACCAATTATCAGCAAACTC-3′ | 5′-TTCTGCTGTATCCTCTTCACCAGT-3′ |
| **CHOP^2^** | 5′-AGAACCAGGAAACGGAAACAGA-3′ | 5′-TCTCCTTCATGCGCTGCTTT-3′ |
| **NOXA**^3^ | 5′-GAGATGCCTGGGAAGAAGG-3′ | 5′-TTCTGCCGGAAGTTCAGTTT-3′ |
| **GAPDH** | 5′-GAAGGTGAAGGTCGGAGT-3′ | 5′-CATGGGTGGAATCATAATGGAA-3′ |
| **TaqMan probes** | | |
| **PROBE** | **(FAM) ID** | **(VIC) ID** |
| **IRE1** | Hs00176385_m1 | GAPDH (4326317E) |
| **ABCB1** | Hs00184500_m1 |  |
| **ABCG2** | Hs01053790_m1 |  |
| **ABCC2** | Hs00166123_m1 |  |

ABCB1: ATP Binding Cassette Subfamily B Member 1; ABCC2: ATP Binding Cassette Subfamily C Member 2; ABCG2: ATP Binding Cassette Subfamily G Member 2; BIP: Binding-Immunoglobulin Protein; CHOP: C/EBP-Homologous Protein; GAPDH: Glyceraldehyde-3-Phosphate Dehydrogenase; IRE1α: Inositol-Requiring Enzyme 1; NOXA: PMAIP1 (Phorbol-12-Myristate-13-Acetate-Induced Protein 1); XBP1: X-Box-Binding Protein 1.

**Supplementary Table S3:** IC_50_ values and 95% confidence intervals (CI) obtained from dose-response curves with PIs bortezomib and carfilzomib in breast cancer cell lines and patient-derived primary cells.

|  | BTZ | | CFZ | |
| --- | --- | --- | --- | --- |
| Cells | **IC_50_ (nM)** | **95% CI** | **IC_50_ (nM)** | **95% CI** |
| MDA-MB-231 | 374.3 | 276.1 – 507.5 | 266.3 | 190.2 – 373.0 |
| BT549 | 346.0 | 297.2 - 402.9 | 257.0 | 217.6 – 303.7 |
| MCF-7 | 3491 | 2571 – 4738 | 2563 | 1794 – 3622 |
| BT474 | 3183 | 2099 – 4828 | 1626 | 1215 – 2175 |
| BCa12 | 114.3 | 70.18 – 186.3 | 228.8 | 135.3 – 387.1 |
| BCa14 | 173.2 | 120.7 – 248.4 | 344.7 | 211.1 – 562.7 |
| BCa15 | 167.3 | 112.5 – 248.8 | 252.1 | 156.2 – 407.0 |

BTZ: bortezomib; CFZ: carfilzomib; CI: confidence interval

**Supplementary Table S4:** IC_50_ values of bortezomib and carfilzomib in cell lines and the ratio of spliced to unspliced XBP1.

BTZ: bortezomib; CFZ: carfilzomib

| **Cell line** | **IC50 (nM) BTZ** | **IC50 (nM) CFZ** | **s/uXBP1** |
| --- | --- | --- | --- |
| AMO-1 | 2.89 | 5.17 | 0.44 |
| AMO-CFZ | 48.71 | 620.00 | 0.13 |
| H460 | 175.20 | 245.70 | 0.11 |
| RPMI-8226 | 5.60 | 6.03 | 0.11 |
| ARH-BTZ | 164.06 | 30.90 | 0.10 |
| ARH-77 | 12.13 | 2.82 | 0.10 |
| HBL1 | 6.68 | 4.03 | 0.09 |
| U2OS | 27.95 | 40.23 | 0.09 |
| MDA-MB-231 | 24.43 | 29.73 | 0.08 |
| AMO-BTZ | 248.10 | 44.55 | 0.07 |
| L363 | 5.28 | 1.82 | 0.05 |
| Colo357 | 47.38 | 54.95 | 0.05 |
| A549 | 613.70 | 1054.00 | 0.04 |
| HEK293T | 106.40 | 131.30 | 0.04 |
| Caki-1 | 1857.00 | 3804.00 | 0.04 |
| MM1R | 2.69 | 1.05 | 0.04 |
| MM1S | 3.57 | 1.76 | 0.04 |
| SK-N-AS | 19.43 | 24.34 | 0.03 |
| HeLa | 22.69 | 92.56 | 0.03 |
| U251 | 206.20 | 337.60 | 0.03 |
| LN229 | 25.37 | 26.17 | 0.02 |
| Hep2G | 128.40 | 218.40 | 0.02 |
| HT1080 | 37.54 | 124.60 | 0.02 |
| BT549 | 31.60 | 136.90 | 0.02 |
| MiaPaCa | 551.00 | 793.80 | 0.01 |
| Panc-1 | 112.00 | 310.20 | 0.01 |
| MCF-7 | 10343.99 | 7267.00 | 0.00 |
| BT474 | 807.30 | 281.70 | 0.00 |

**Supplementary Table S5:** Fold change of MTG efflux inhibition and fold change of increased cytotoxicity to carfilzomib caused by lopinavir.

| **Cells** | **FC IC_50_ CFZ *vs.* CFZ+LPV** | **FC MTG fluorescence intensity Ctrl vs LPV** |
| --- | --- | --- |
| **MDA-MB-231** | **10.7** | 1.38 |
| **BT549** | **6.1** | 1.19 |
| **MCF-7** | **4.1** | 1.01 |
| **BT474** | **0.8** | 0.85 |
| **BCa12** | **2.43** | 1.09 |
| **BCa14** | **2.61** | 1.07 |
| **BCa15** | **2.66** | 1.22 |

BTZ: bortezomib; CFZ: carfilzomib; FC: fold change; LPV: lopinavir; MTG: Mitotracker Green

**SUPPLEMENTARY FIGURES**


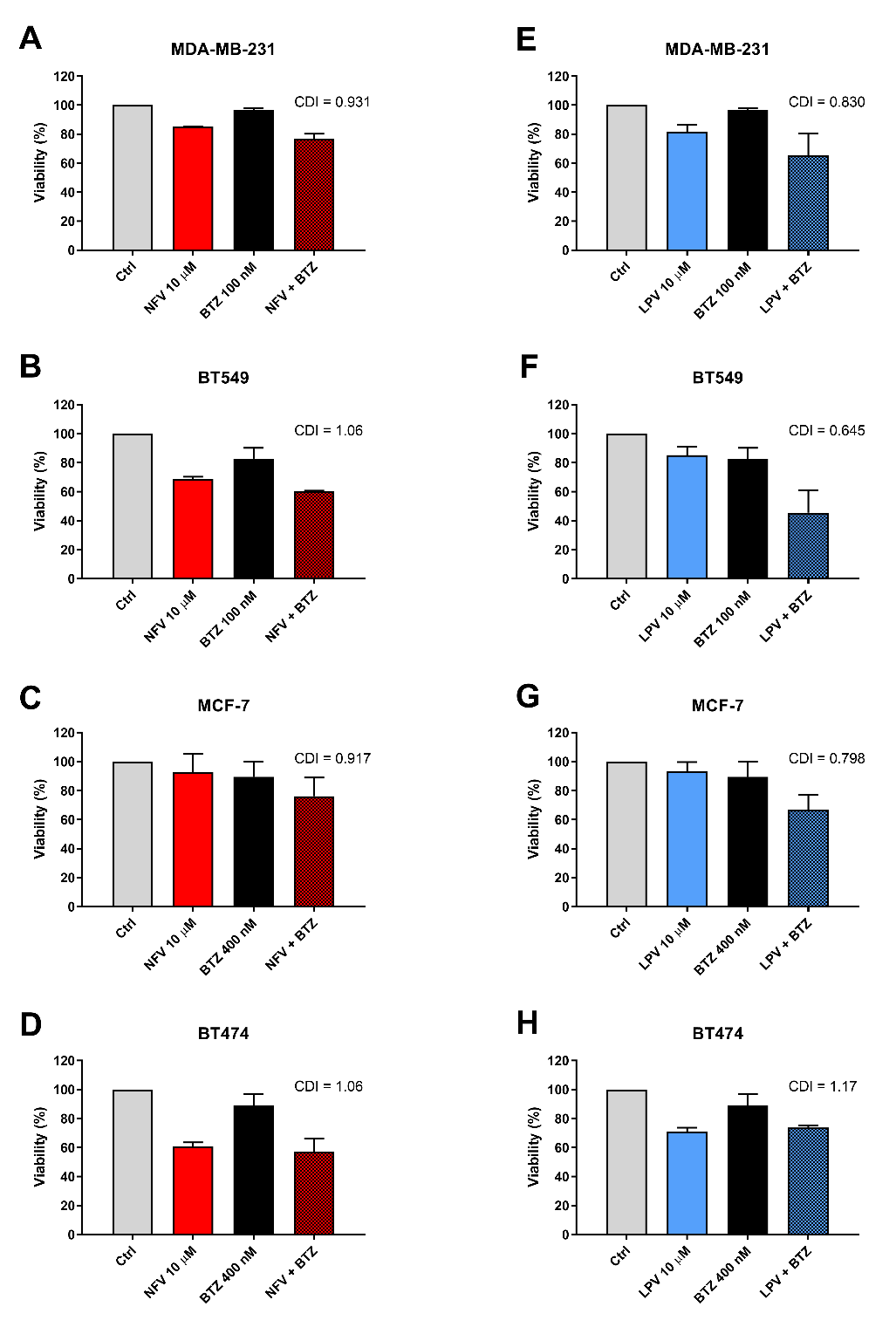


**Supplementary Figure S1: Cytotoxicity of the combination of bortezomib and HIV-protease inhibitors in breast cancer cell lines, selected from Figure 2C.** (A-D) Cytotoxicity of the combination of bortezomib and nelfinavir. (E-H) Cytotoxicity of the combination of bortezomib and lopinavir. For each cell line and treatment, the cytotoxicity of the drugs in monotherapy and in combination is presented. The coefficient of drug interaction (CDI) was calculated and is indicated. The data represent the mean ± SD of at least 3 independent experiments.

BTZ: bortezomib; LPV: lopinavir; NFV: nelfinavir; CDI: coefficient of drug interaction.


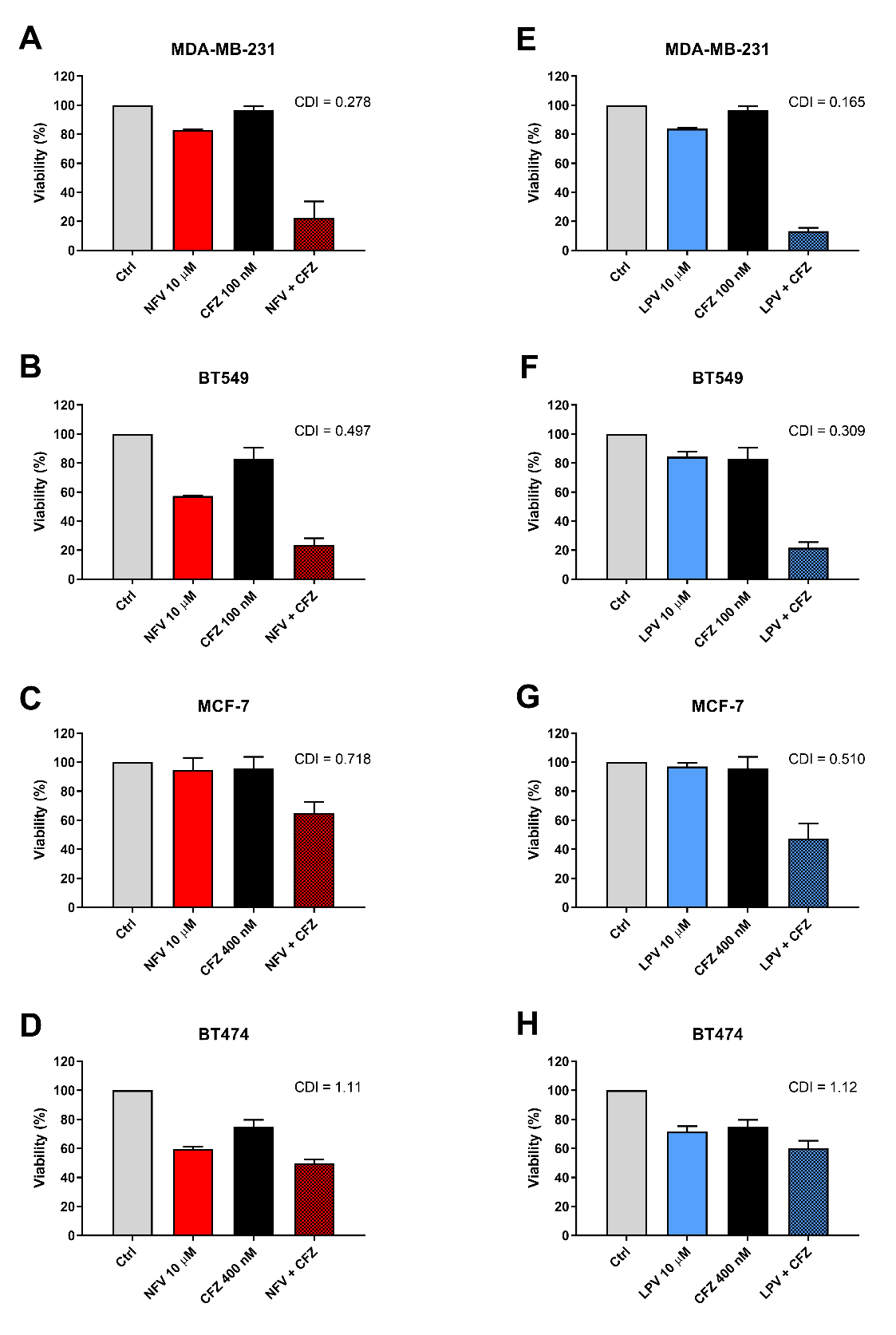


**Supplementary Figure S2: Cytotoxicity of the combination of carfilzomib and HIV-protease inhibitors in breast cancer cell lines, selected from Figure 2D.** (A-D) Cytotoxicity of the combination of carfilzomib and nelfinavir. (E-H) Cytotoxicity of the combination of carfilzomib and lopinavir. For each cell line and treatment, the cytotoxicity of the drugs in monotherapy and in combination is presented. The coefficient of drug interaction (CDI) was calculated and is indicated. The data represent the mean ± SD of at least 3 independent experiments.

CFZ: carfilzomib; LPV: lopinavir; NFV: nelfinavir; CDI: coefficient of drug interaction.


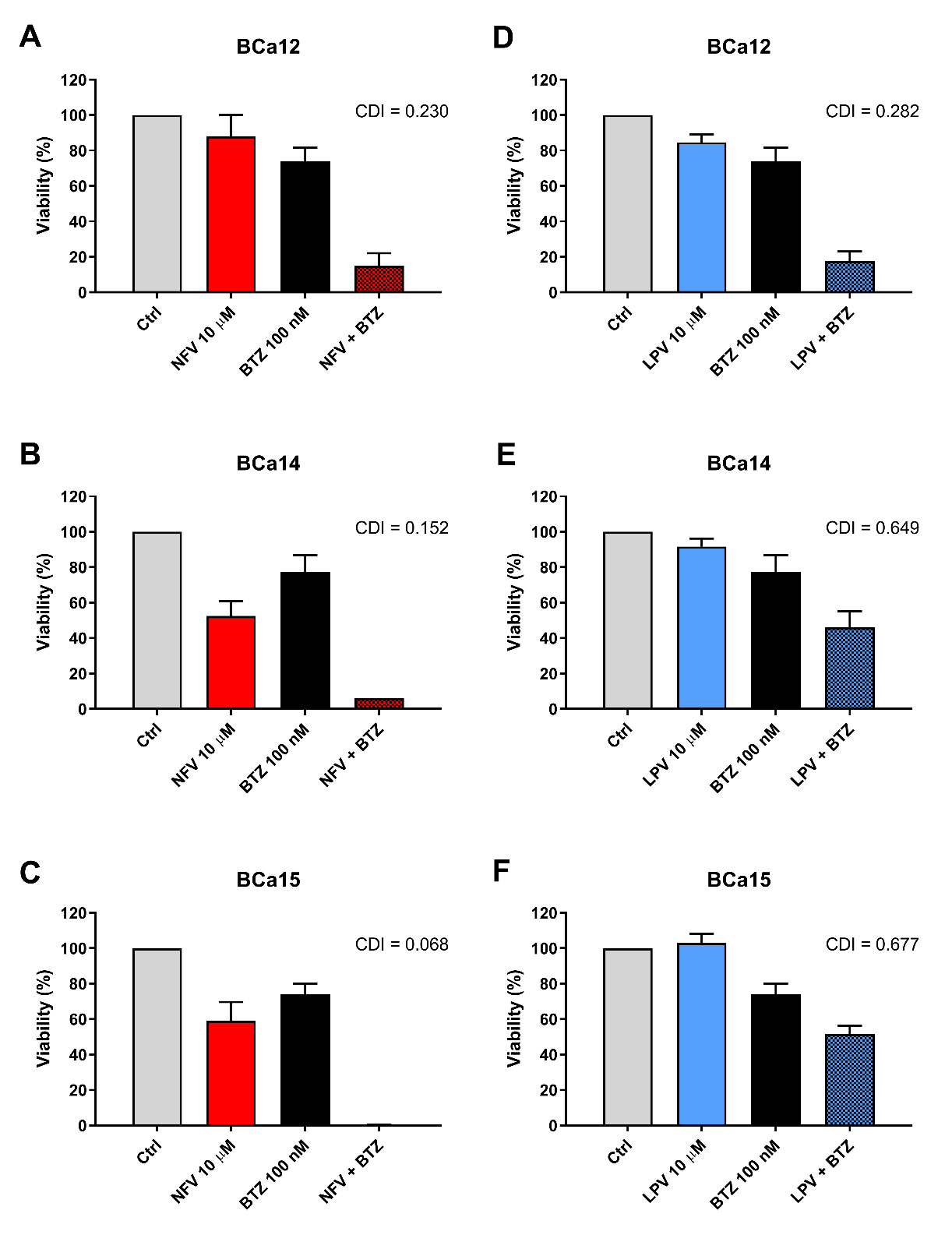


**Supplementary Figure S3: Cytotoxicity of the combination of bortezomib and HIV-protease inhibitors in primary breast cancer cells, selected from Figure 2E.** (A-C) Cytotoxicity of the combination of bortezomib and nelfinavir. (D-F) Cytotoxicity of the combination of bortezomib and lopinavir. For each patient and treatment, the cytotoxicity of the drugs in monotherapy and in combination is presented. The coefficient of drug interaction (CDI) was calculated and is indicated. The data represent the mean ± SD of at least 2 independent experiments.

BTZ: bortezomib; LPV: lopinavir; NFV: nelfinavir; CDI: coefficient of drug interaction.


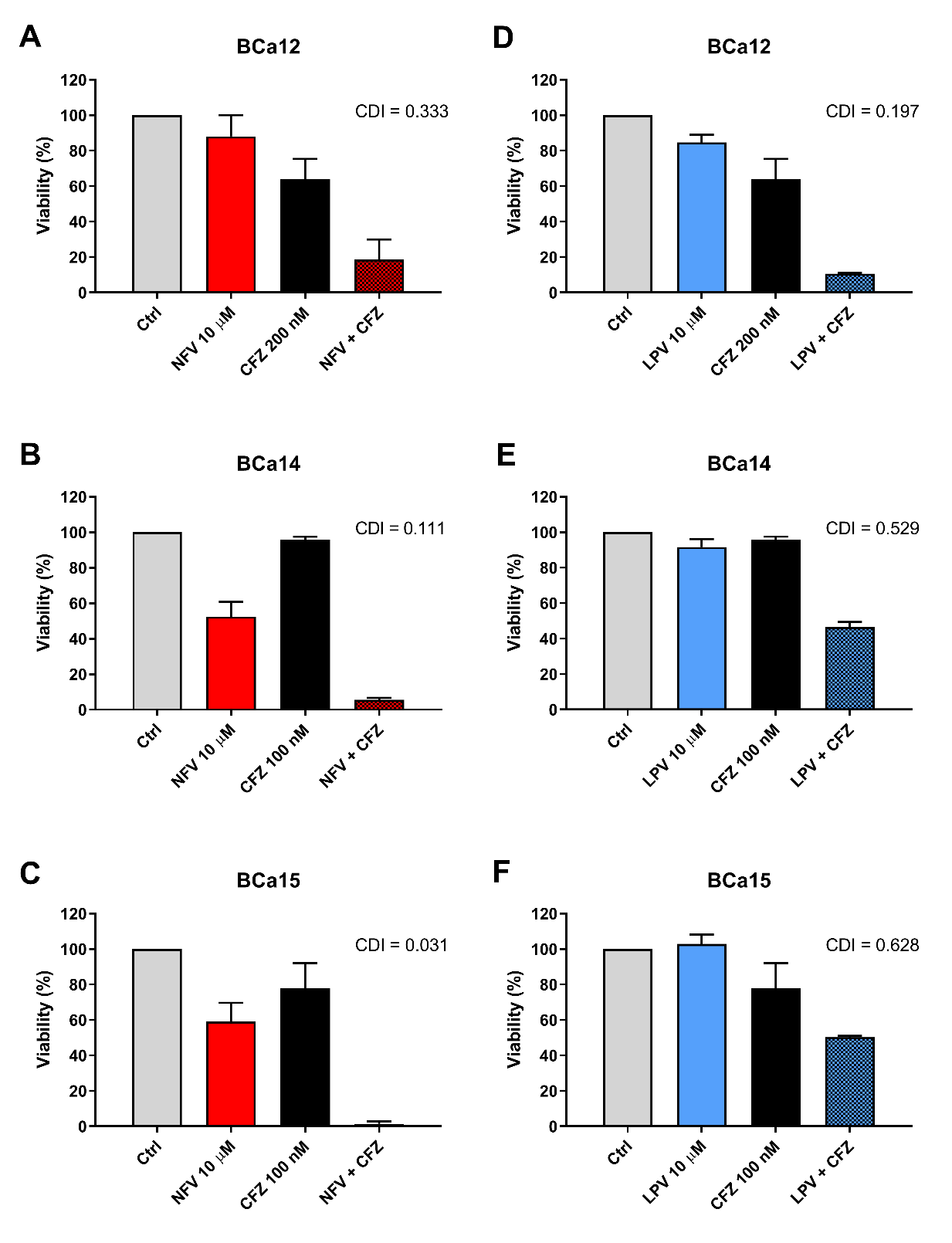


**Supplementary Figure S4: Cytotoxicity of the combination of carfilzomib and HIV-protease inhibitors in primary breast cancer cells, selected from Figure 2F.** (A-C) Cytotoxicity of the combination of carfilzomib and nelfinavir. (D-F) Cytotoxicity of the combination of carfilzomib and lopinavir. For each cell line and treatment, the cytotoxicity of the drugs in monotherapy and in combination is presented. The coefficient of drug interaction (CDI) was calculated and is indicated. The data represent the mean ± SD of at least 2 independent experiments.

CFZ: carfilzomib; LPV: lopinavir; NFV: nelfinavir; CDI: coefficient of drug interaction


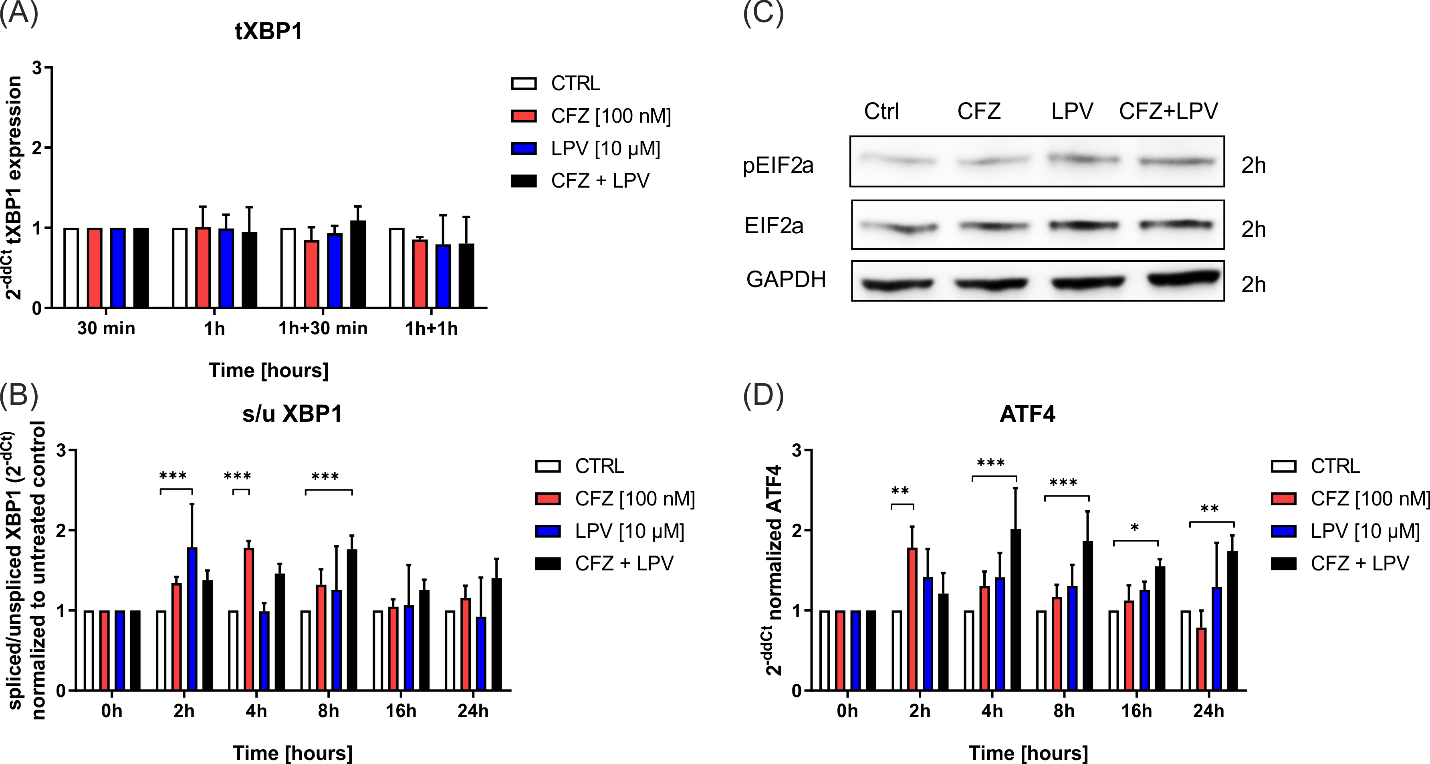


**Supplementary Figure S5: Analysis of the UPR induction in MDA-MB-231 by the combination treatment.**

(A) Total XBP1 expression determined by qPCR, normalized to GAPDH and a time-point 30 min before treatment with carfilzomib, lopinavir or their combination. (B) Induction of spliced XBP1(sXBP1), determined by qPCR and presented as a ratio of spliced vs. unspliced XBP1 RNA variants normalized to GAPDH and a time-point of 0 h after the 1 h pulse treatment with carfilzomib or continuous treatment with lopinavir. (C) Representative western blot image of EIF2α phosphorylation, represented by a phosphorylated and total form of eIF2α 2 h after the 1 h pulse treatment with carfilzomib or continuous treatment with lopinavir. (D) ATF4 expression determined by qPCR, normalized to GAPDH and a time-point 0 h after the 1 h pulse treatment with carfilzomib or continuous treatment with lopinavir. In all qPCR experiments, the data represent the mean ± SD of 3 independent experiments. Statistical significance was determined using one-way ANOVA with Tukey post-test. * represents p < 0.05, *** represents p < 0.001.

ATF4: Activating Transcription Factor 4; CFZ: carfilzomib; EIF2α: Eukaryotic Translation Initiation Factor 2A; GAPDH: Glyceraldehyde-3-Phosphate Dehydrogenase; LPV: lopinavir; XBP1: X-Box-Binding Protein 1

**
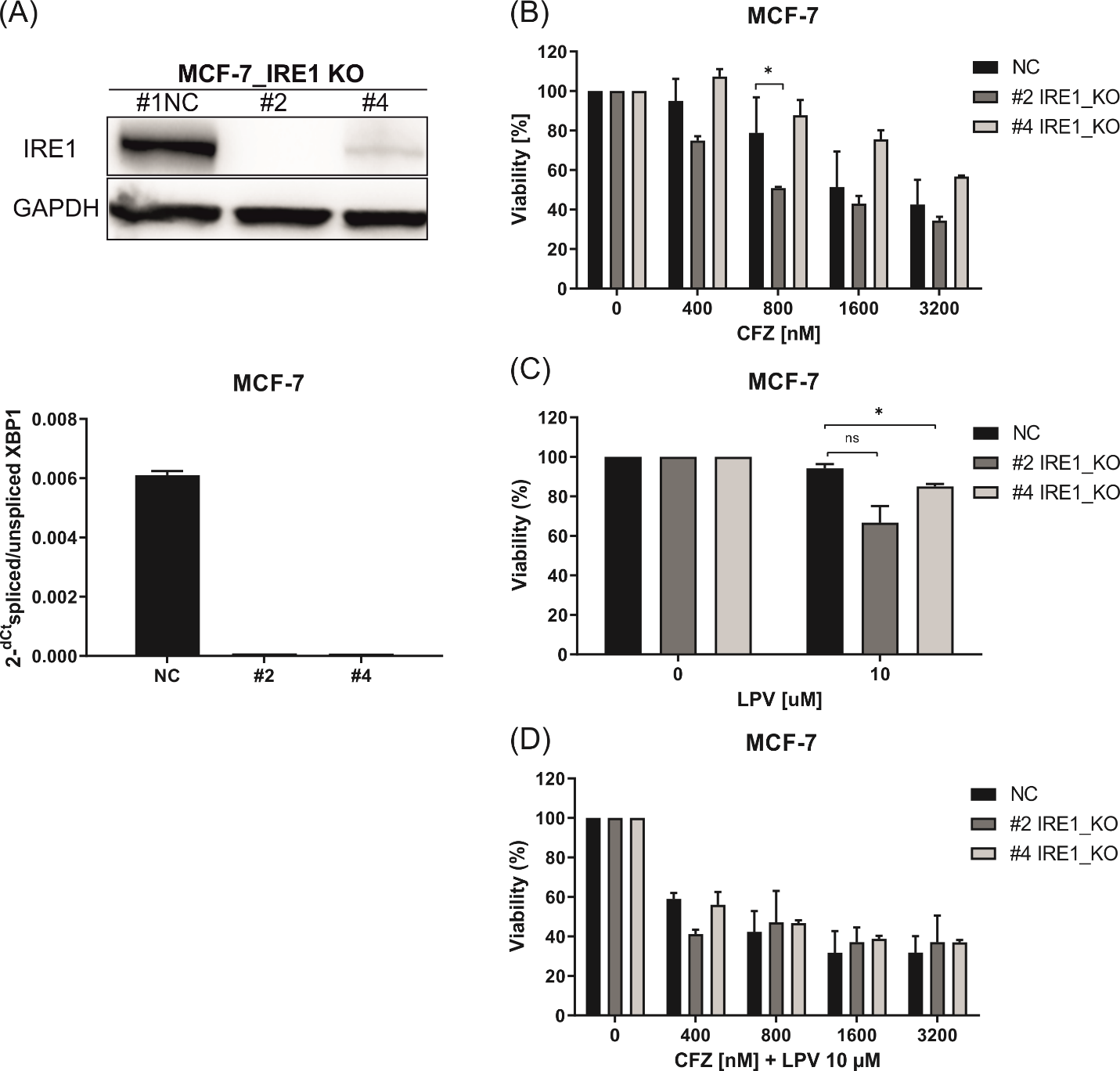
**

**Supplementary Figure S6: Role of sXBP1 in monotherapy with carfilzomib and lopinavir, or in drug combination in MCF-7 cells.**

(A) Representative western blot image of IRE1α knock-out in control or in two single-cell derived clones #2 and #4 of MCF-7 cell line (upper part), leading to functional decrease of sXBP1 evaluated by qPCR, assessed as a ratio of spliced vs. unspliced XBP1 and normalized to GAPDH, which served as a housekeeping gene (bottom part). (B) Cytotoxicity of carfilzomib in MCF-7 cells with normal level of IRE1α (NC) or in single-cell derived clones (#2 and #4) with knocked-out IRE1α. Viability was assessed after 1 h of pulse treatment and continuous 48 h incubation in drug-free medium. The data represent the mean ± SD of 3 independent experiments. Statistical significance was determined using unpaired t-test. (C) Cytotoxicity of lopinavir in MCF-7 cells with normal level of IRE1α (NC) or in single-cell-derived clones (#2 and #4) with knocked-out IRE1α. Viability was assessed after 48 h of continuous treatment. The data represent the mean ± SD of 3 independent experiments. Statistical significance was determined using unpaired t-test. (D) Cytotoxicity of carfilzomib and lopinavir combination in MCF-7 cells with normal level of IRE1α (NC) or in single-cell derived clones (#2 and #4) with knocked-out IRE1α. Viability was assessed after 1 h pulse treatment with carfilzomib and continuous 48 h treatment with lopinavir. The data represent the mean ± SD of 3 independent experiments. Statistical significance was determined using unpaired t-test.

CFZ: carfilzomib; GAPDH: Glyceraldehyde-3-Phosphate Dehydrogenase; IRE1α: Inositol-Requiring Enzyme 1; LPV: lopinavir; XBP1: X-Box-Binding Protein 1


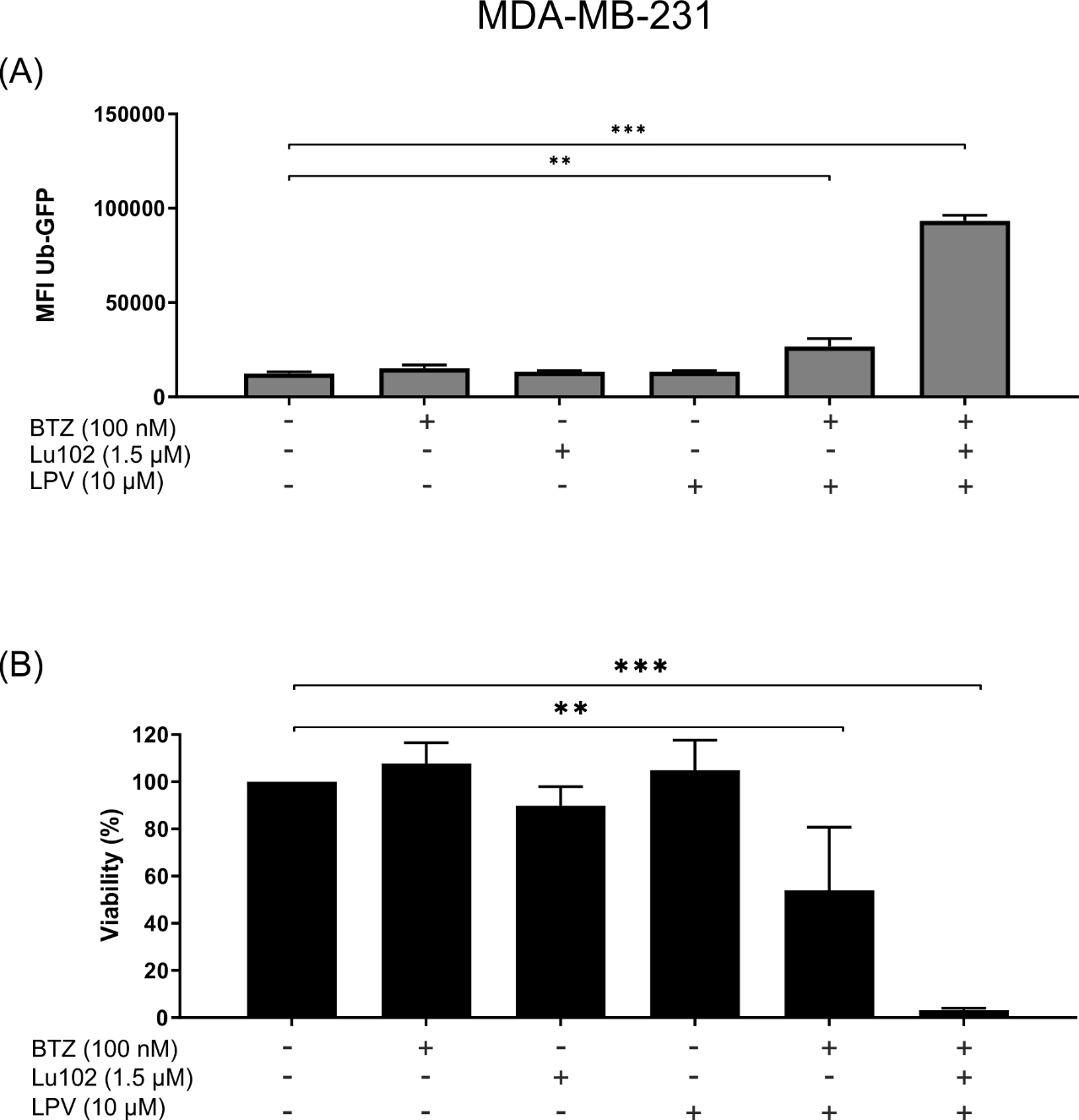


**Supplementary Figure S7: Effect of bortezomib co-treatment with LU102 and lopinavir on proteasome activity and viability in MDA-MB-231 cells stably expressing Ub^G76V^-GFP.**

(A) Median fluorescence intensity (MFI) of Ub^G76V^-GFP, corresponding to functional proteasome inhibition in the cells. The cells were treated with bortezomib, LU102 or their combination for 1 h and subsequently placed in drug-free medium or into medium containing 10 µM lopinavir for 8 h. The data represent the mean ± SD of 3 independent experiments. Statistical significance was determined using unpaired t-test, ** represents p < 0.01; *** represents p < 0.001. (B) Viability corresponding to (A). The cells were treated with bortezomib, LU102 or their combination for 1h and subsequently placed in drug-free medium or in medium with lopinavir for 48 h. The data represent the mean ± SD of 3 independent experiments. Statistical significance was determined using unpaired t-test, ** represents p < 0.01; *** represents p < 0.001.

BTZ: bortezomib; LPV: lopinavir; LU102: β2 specific inhibitor; MFI: median fluorescence intensity; Ub^G76V^-GFP: mutated uncleavable ubiquitin moiety – green fluorescent protein

**
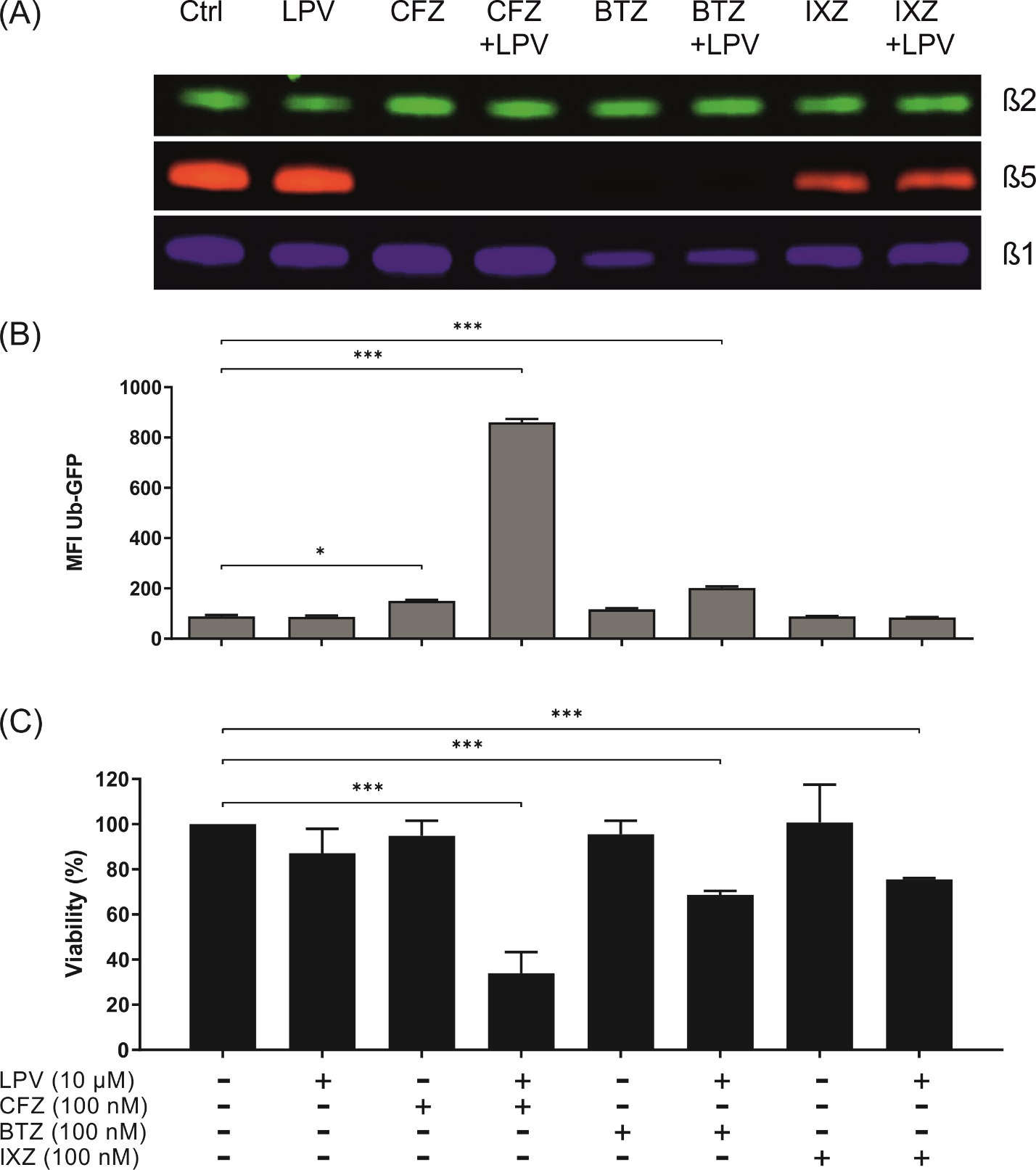
**

**Supplementary Figure S8: Effect of the clinically approved proteasome inhibitors combined with lopinavir on proteasome activity and viability in MDA-MB-231 cells stably expressing Ub^G76V^-GFP.**

(A) Representative gel image of residual activity of the proteasome β2, β5 and β1 subunits, visualized by ABP labeling 1 h after treatment. The cells were treated with carfilzomib, bortezomib and ixazomib as monotherapy or in combination with lopinavir for 1 h and subsequently lysed and incubated with ABP. (B) Median fluorescence intensity (MFI) of Ub^G76V^-GFP, corresponding to functional proteasome inhibition in the cells. The cells were treated with carfilzomib, bortezomib or ixazomib in monotherapy for 1 h and subsequently placed in drug-free medium or into medium containing 10 µM lopinavir for 8 h. The data represent the mean ± SD of 3 independent experiments. Statistical significance was determined using unpaired t-test, ** represents p < 0.01; *** represents p < 0.001. (C) Viability corresponding to (A) and (B). The cells were treated with carfilzomib, bortezomib or ixazomib for 1h and subsequently placed in drug-free medium or in medium with lopinavir for 48 h. The data represent the mean ± SD from 3 independent experiments. Statistical significance was determined using unpaired t-test, ** represents p < 0.01; *** represents p < 0.001.

BTZ: bortezomib; CFZ: carfilzomib; IXA: ixazomib; LPV: lopinavir; MFI: median fluorescence intensity; Ub^G76V^-GFP: mutated uncleavable ubiquitin moiety – green fluorescent protein

**
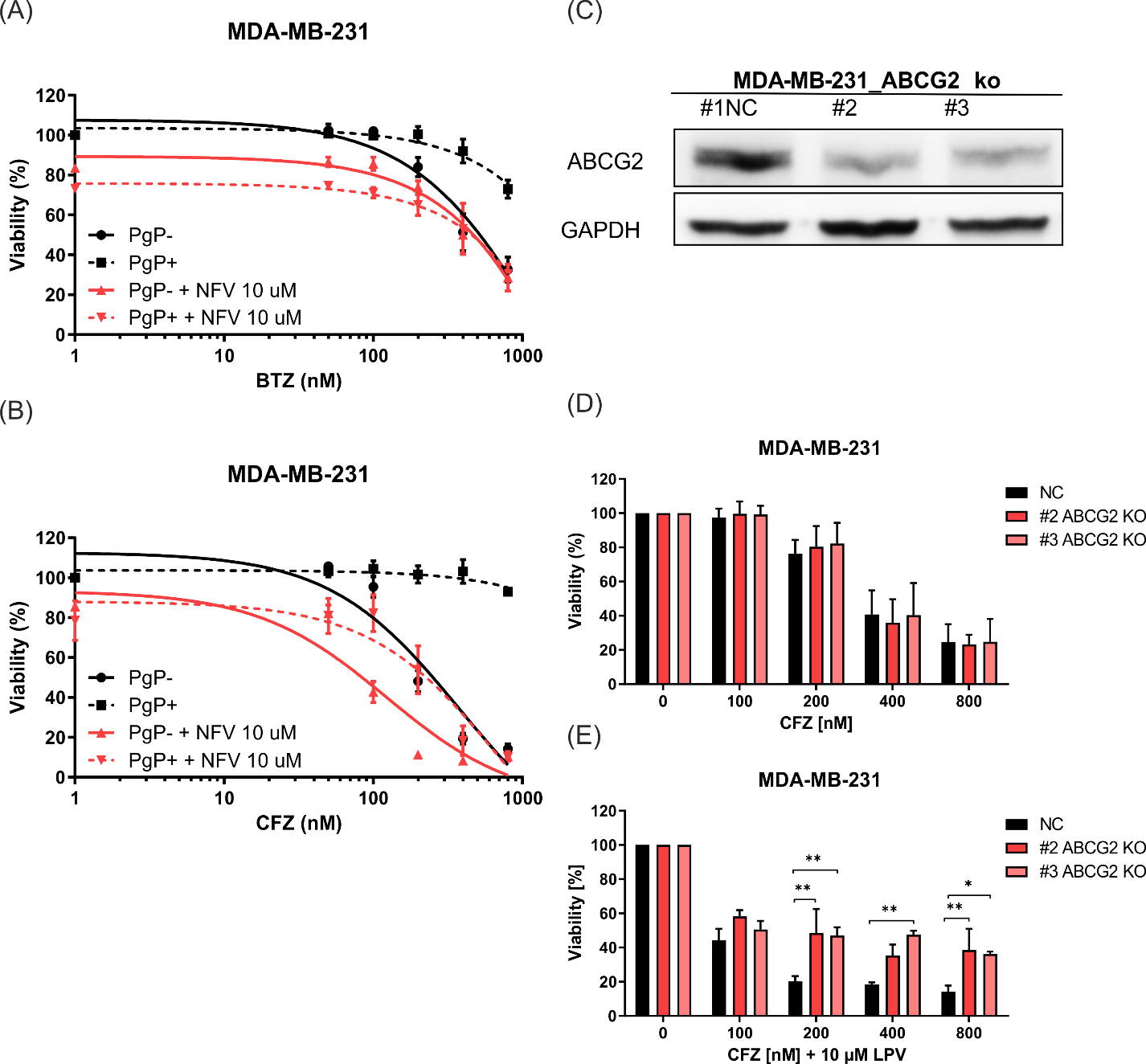
**

**Figure S9: Role of ABCB1 and ABCG2 in the cytotoxicity of carfilzomib in monotherapy or in combination with lopinavir in MDA-MB-231 cells.**

(A-B) Dose-response curves of MDA-MB-231 cells without PgP (PgP-) or with introduced PgP (PgP+) to bortezomib (A) and carfilzomib (B) in monotherapy or in combination with nelfinavir. The cells were treated with proteasome inhibitors for 1 h and subsequently placed in drug-free medium or in medium with nelfinavir for 48 h. The data represent the mean ± SD of 3 independent experiments. (C) Representative western blot image of ABCG2 knock-out in control or in two single-cell-derived clones #2 and #3 of MDA-MB-231 cell line. GAPDH serves as a loading control. (D) Cytotoxicity of carfilzomib in MDA-MB-231 cells with normal level of ABCG2 (NC) or in single-cell derived clones (#2 and #3) with knocked-out ABCG2. Viability was assessed after 1 h pulse treatment with carfilzomib and continuous 48 h incubation in drug-free medium. The data represent the mean ± SD from 3 independent experiments. Statistical significance was determined with unpaired t-test. (E) Cytotoxicity of carfilzomib and lopinavir combination in MDA-MB-231 cells with normal level of ABCG2 (NC) or in single-cell-derived clones (#2 and #3) with knocked-out ABCG2. Viability was assessed after 1 h pulse treatment with carfilzomib and continuous 48 h treatment with lopinavir. The data represent the mean ± SD of 3 independent experiments. Statistical significance was determined using unpaired t-test, * represents p < 0.05; ** represents p < 0.01.

ABCG2: ATP Binding Cassette Subfamily G Member 2; CFZ: carfilzomib; GAPDH: Glyceraldehyde-3-Phosphate Dehydrogenase; LPV: lopinavir

**REFERENCES**

1. Dantuma NP, Lindsten K, Glas R, Jellne M, Masucci MG. Short-lived green fluorescent proteins for quantifying ubiquitin/proteasome-dependent proteolysis in living cells. Nat Biotechnol. 2000;18(5):538-43.

2. Merksamer PI, Trusina A, Papa FR. Real-time redox measurements during endoplasmic reticulum stress reveal interlinked protein folding functions. Cell. 2008;135(5):933-47.

3. Kanekura K, Ishigaki S, Merksamer PI, Papa FR, Urano F. Establishment of a system for monitoring endoplasmic reticulum redox state in mammalian cells. Lab Invest. 2013;93(11):1254-8.

4. Lai CW, Aronson DE, Snapp EL. BiP availability distinguishes states of homeostasis and stress in the endoplasmic reticulum of living cells. Mol Biol Cell. 2010;21(12):1909-21.

5. Sanjana NE, Shalem O, Zhang F. Improved vectors and genome-wide libraries for CRISPR screening. Nat Methods. 2014;11(8):783-4.

6. Shalem O, Sanjana NE, Hartenian E, Shi X, Scott DA, Mikkelsen TS, et al. Genome-scale CRISPR-Cas9 knockout screening in human cells. Science. 2014;343(6166):84-7.
